# Modular integration of nanopore sequencing, alphafold modeling, and statistical design boosts 1-hydroxyphenazine yield in *Pseudomonas aeruginosa* strain KAEH25

**DOI:** 10.1186/s12866-026-05221-2

**Published:** 2026-07-13

**Authors:** Mahmoud Abd El-Mongy, Khaled Abuelhaded, Adel AbdelKhalek, Ahmed Hassan Ibrahim Faraag

**Affiliations:** 1 Department of Microbial Biotechnology, Genetic Engineering and Biotechnology Research Institute, University of Sadat City, Sadat City, Egypt; 2 School of Biotechnology, Badr University in Cairo (BUC), Badr, 11829 Egypt; 3 Food Safety, Hygiene and Technology Department, Faculty of Veterinary Medicine, Badr University in Cairo (BUC), Badr, 11829 Egypt; 4 Botany and Microbiology Department, Faculty of Science, Capital University (formerly Helwan University), Cairo, Egypt; 5 Medical Biotechnology Department, School of Biotechnology, Badr University in Cairo (BUC), Badr, 11829 Egypt

**Keywords:** *Pseudomonas aeruginosa*, 1-hydroxyphenazine, Nanopore sequencing, AlphaFold, Plackett–Burman, Response surface methodology, Phenazine biosynthesis

## Abstract

This study establishes an integrated genome-to-structure-to-process framework that significantly enhances 1-hydroxyphenazine (1-OH-PHZ) biosynthesis in *Pseudomonas aeruginosa* KAEH25. Nanopore long-read sequencing completely resolved the 10,358 bp phenazine biosynthetic locus, confirming intact organization of core (*phzB–phzG*) and tailoring (*phzH*, *phzS*) genes. AlphaFold modeling and InterProScan analysis validated conserved catalytic domains across all pathway enzymes, confirming functional coherence. Plackett–Burman screening identified temperature, pH, glucose, peptone, inoculum size, and incubation time as significant production determinants, while response surface methodology optimized these factors to achieve a maximum 1-OH-PHZ titre of 24.85 µg mL⁻^1^ under refined conditions (37 °C, pH 6.5–7, 1% glucose, 2 g L⁻^1^ peptone, 10 mL inoculum, 48 h). The statistical model demonstrated exceptional explanatory power (R^2^ = 0.9605; Adeq Precision = 19.11). Preliminary antimicrobial assays demonstrated selective activity of 1-hydroxyphenazine (1-OH-PHZ), with inhibition zones of 20 ± 1 mm against *Escherichia coli*, 13 ± 0.5 mm against *Salmonella enterica* serovar Typhimurium, 13 ± 0.7 mm against *Klebsiella pneumoniae*, and 8 ± 0.3 mm against *Staphylococcus aureus*, while no inhibition was observed against *Enterococcus faecalis* or *Listeria monocytogenes*. Collectively, this systems-guided approach provides a transferable platform for optimizing microbial secondary metabolites and validates 1-OH-PHZ as a promising lead for antimicrobial development.

**Supplementary Information:**

The online version contains supplementary material available at 10.1186/s12866-026-05221-2.

## Introduction

*Pseudomonas aeruginosa* is a Gram-negative bacterium with broad metabolic adaptability and the ability to persist in diverse environments [1]. It is also a significant opportunistic pathogen, causing severe infections in immunocompromised individuals [2]. Among its diverse secondary metabolites, 1-hydroxyphenazine (1-OH-PHZ) is a redox-active virulence factor that generates reactive oxygen species, contributing to host tissue damage [3]. Beyond its role in pathogenesis, 1-OH-PHZ exhibits broad-spectrum antimicrobial activity through mechanisms such as redox cycling leading to oxidative damage, disruption of microbial cell membranes, inhibition of DNA/RNA synthesis, interference with biofilm formation, and potential synergy with other antibiotics [4–6]. Despite its importance, the production titers of 1-OH-PHZ remain suboptimal for both mechanistic studies and biotechnological applications [7, 8].

The biosynthesis of phenazines, including 1-OH-PHZ, is directed by the *phz* operon (comprising *phzA-G* and accessory genes *phzH*, *phzM*, and *phzS*) which reroutes chorismate from the shikimate pathway into specialized phenazine intermediates [9]. Specifically, PhzE converts chorismate to 2-amino-2-deoxyisochorismate (ADIC), which PhzD cleaves to yield *trans*−2,3-dihydro-3-hydroxyanthranilate. PhzF facilitates a tautomerization step, followed by PhzA and PhzB collaborating to construct the phenazine scaffold. PhzG then catalyzes the oxidative aromatization to produce phenazine-1-carboxylic acid (PCA) [10]. Tailoring enzymes modify PCA: PhzH amidates PCA to phenazine-1-carboxamide (PCN), while PhzS, a flavin-dependent monooxygenase, hydroxylates PCA to 1-OH-PHZ. The methyltransferase PhzM, in conjunction with these reactions, can also lead to pyocyanin formation [7]. Disruptions in these core or accessory genes can significantly impact overall phenazine production [9]. The catalytic efficiency of enzymes like PhzO, responsible for 2-hydroxyphenazine production, has been identified as a limiting factor, often showing conversion rates as low as 10%–20%, which limits the yield of hydroxylated phenazines [11, 12].

Current limitations in enhancing 1-OH-PHZ production stem from several knowledge gaps. Empirical screening approaches for process optimization often lack integration with genomic or structural insights, leading to fragmented and non-transferable strategies [7, 13]. Specifically for *P. aeruginosa* strain KAEH25, while the core *phz* operon and key tailoring enzymes like PhzS are identified, strain-specific genomic features, such as structural variants, regulatory mutations, or cryptic accessory genes that influence 1-OH-PHZ flux, remain largely uncharacterized [14, 15]. Furthermore, the detailed structural basis for the catalytic efficiency and substrate specificity of critical pathway enzymes, such as PhzS, is not fully understood, hindering rational engineering efforts [11, 16].

This study addresses these limitations by integrating a genome-to-structure-to-process workflow to enhance 1-OH-PHZ production in *P. aeruginosa* strain KAEH25. The methodology employs Nanopore long-read sequencing to generate a highly accurate, detailed genome assembly, facilitating the identification of genes and genomic features crucial for 1-OH-PHZ biosynthesis, particularly in complex or repetitive regions and for resolving structural variants [17]. This genomic analysis will uncover potential mutations affecting product yield and identify strain-specific elements [18]. Concurrently, AlphaFold, leveraging deep neural networks, will predict the three-dimensional structures of the pathway's enzymes with high precision, providing critical insights into their catalytic mechanisms, cofactor requirements, and potential bottlenecks [19–22].

These structural insights, combined with genomic data, will inform the rational selection of optimal culture medium components and growth conditions to maximize enzyme activity and 1-OH-PHZ production [12]. Finally, a Plackett–Burman statistical experimental design will be utilized to efficiently screen multiple cultivation parameters, identifying those with significant effects on 1-OH-PHZ yield, such as pH, temperature, nutrient concentrations, and aeration rates [17, 23]. This initial screening will be followed by more refined optimization techniques like RSM [8, 12].

This study aims to establish an integrated, predictive framework that unites genome mining, structural biology, and statistical process optimization to rationally enhance the biosynthesis of 1-OH-PHZ in *P.aeruginosa* KAEH25. By resolving the complete phenazine biosynthetic locus through long-read sequencing, elucidating the structural and functional features of key pathway enzymes using AlphaFold-based modeling, and identifying the most influential cultivation parameters through Plackett–Burman and response-surface methodologies, the study seeks to overcome existing gaps in genetic characterization, enzyme-level understanding, and culture-condition optimization. Ultimately, the goal is to develop a systems-level strategy that accelerates 1-OH-PHZ production and provides a transferable platform for improving the yield of other microbial secondary metabolites within contemporary synthetic-biology and industrial-biotechnology frameworks.

## Methods

### Bacterial sample collection and screening

Rhizospheric soil was collected from four-week-old barley (*Hordeum vulgare* L. ‘Giza 132’) grown in Menofia Governorate, Egypt [24]. Sampling was performed at a depth of 5–15 cm using ethanol-sterilized stainless-steel spoons. Approximately 1–2 g of soil was transferred into sterile 50 mL DNA/RNA-free polypropylene tubes and transported on ice (< 4 °C). Samples were processed within 2 h of collection. For homogenization, 1 g of soil was suspended in 9 mL sterile phosphate-buffered saline (PBS, pH 7.2) and vortexed thoroughly. Serial ten-fold dilutions (10⁻^1^–10⁻^4^) were prepared in sterile PBS. From each dilution, 100 µL aliquots were spread-plated in duplicate onto Reasoner’s 2 A (R2A) agar and King’s B agar. Plates were incubated aerobically at 28 ± 1 °C for 48–72 h. Colonies exhibiting orange-red pigmentation were streak-purified twice on Luria–Bertani (LB) agar to ensure clonal isolation. Pigment-producing isolates were screened using (i) visual inspection under ambient light, (ii) UV trans-illumination at 365 nm to detect characteristic orange fluorescence, and (iii) spectrophotometric measurement of pigment extracts at 367 nm to confirm phenazine production [25–29].

### Bacterial strain and culture conditions

*Pseudomonas aeruginosa* KAEH25 was maintained on Nutrient Agar (NA) plates solidified with 1.5% Bacto™ Agar and stored at 4 °C for short-term preservation. For seed culture preparation, a single isolated colony was inoculated into 5 mL Lysogeny Broth (LB) and incubated at 37 ± 1 °C with orbital shaking at 180 rpm until the culture reached an optical density at 600 nm (OD₆₀₀) of 0.50 ± 0.02 (18–24 h). The resulting pre-culture was diluted 1:100 (v/v) into 50 mL fresh LB medium and incubated again under identical temperature and agitation conditions until late-log-phase biomass was obtained (OD₆₀₀ ≈ 1.0). Cultures were monitored spectrophotometrically at regular intervals to ensure consistent growth prior to downstream phenotypic and molecular assays [30–32]. *Pseudomonas aeruginosa* strain KAEH25 was handled under Biosafety Level 2 (BSL-2) containment throughout all experimental procedures, following institutional and national guidelines for working with opportunistic human pathogens. All experimental procedures were conducted exclusively under in vitro conditions, including bacterial cultivation, genomic characterization, metabolite extraction, and antimicrobial susceptibility testing. No in vivo experiments, environmental release, or clinical applications were performed. All work was carried out within a certified Biosafety Level 2 (BSL-2) facility, following institutional and national biosafety guidelines. Accordingly, all downstream risks were fully contained within standard BSL-2 operational boundaries.

### DNA extraction

High-molecular-weight genomic DNA was extracted from freshly cultured bacterial pellets (≈10⁹ CFU) using the DNeasy® Blood & Tissue Kit (Qiagen GmbH, Hilden, Germany, Cat. No. 69504) following the manufacturer’s Gram-negative protocol (version 10/2024). Pellets were resuspended in 180 µL Buffer ATL, supplemented with 20 µL Proteinase K (≥ 600 mAU mL⁻^1^), and incubated at 56 °C with agitation at 900 rpm for 30 min in a ThermoMixer® C. After adding 200 µL Buffer AL, samples were incubated at 70 °C for 10 min and subsequently transferred to DNeasy Mini spin columns placed in 2 mL collection tubes. Columns were centrifuged at 6,000 × g for 1 min, washed with 500 µL Buffer AW1 and 600 µL Buffer AW2 (8,000 × g, 1 min each), and eluted with 200 µL pre-warmed (65 °C) Buffer AE following a 5-min membrane incubation and centrifugation at 8,000 × g for 1 min. DNA concentration and purity (A₂₆₀/₂₈₀ and A₂₆₀/₂₃₀ ratios) were measured in triplicate using a NanoDrop™ 2000c UV–Vis spectrophotometer calibrated with 1 × TE buffer, yielding 40–60 ng µL⁻^1^ DNA suitable for downstream analyses. For phylogenetic analysis, 16S rRNA gene sequences from *Pseudomonas aeruginosa* KAEH25 and reference strains were aligned using the MUSCLE algorithm. The ProscanGTR + G + I substitution model was selected, and a maximum likelihood phylogenetic tree was generated in Geneious Prime® v.2025.0.5 using 1,000 bootstrap replicates. Branch lengths were calculated using a genetic distance scale of 0.02 substitutions per site.

### Molecular characterization of bacterial strain using 16 s rRNA

*P. aeruginosa* strain KAEH25 was resuscitated from − 80 °C glycerol stocks by streaking onto LB agar (Lennox formulation, 1.5% Bacto™ agar, BD Franklin Lakes, NJ, USA) and incubating aerobically at 37 ± 1 °C for 18 h in a Heratherm™ Compact incubator (Thermo Fisher Scientific, Waltham, MA, USA). A single, well-isolated colony was harvested and genomic DNA extracted with the DNeasy® Blood & Tissue Kit (Qiagen GmbH, Hilden, Germany, Cat. No. 69506) following the manufacturer’s Gram-negative protocol, yielding 30–50 ng µL⁻^1^ high-molecular-weight DNA with A₂₆₀/₂₈₀ ratios of 1.85–2.0 (NanoDrop™ One, Thermo Fisher). The full-length 16S rRNA gene was amplified in 25 µL reactions containing 1 × EmeraldAmp® GT PCR Master Mix (Takara Bio Inc., Shiga, Japan, Cat. No. RR310A), 0.2 µM each of universal primers 27 F (5ʹ-AGAGTTTGATCMTGGCTCAG-3ʹ) and 1492R (5ʹ-TACGGYTACCTTGTTACGACTT-3ʹ) (synthesised by Eurofins Genomics, Ebersberg, Germany), and ~ 20 ng template DNA. Thermal cycling was performed on a T100™ Thermal Cycler (Bio-Rad Laboratories, Hercules, CA, USA) programmed for initial denaturation at 94 °C for 3 min, followed by 30 cycles of 94 °C for 30 s, 55 °C for 30 s, 72 °C for 90 s, and a final extension at 72 °C for 5 min. Amplicons were purified with the QIAquick® PCR Purification Kit (Qiagen; Cat. No. 28104) and bidirectionally sequenced on an ABI 3730xl DNA Analyzer (Macrogen Inc., Daejeon, Republic of Korea). Forward and reverse reads were imported into Geneious Prime® v.2025.0.5 (Biomatters Ltd., Auckland, New Zealand), quality-trimmed (Q ≥ 30), and assembled into a 1,524-bp contig corresponding to the complete 16S rRNA gene. The high-quality consensus sequence was deposited in the NCBI GenBank database under accession number PX255551 [33–36].

### Nanopore DNA sequencing and bioinformatics for characterization of 1‑OH‑PHZ Genes

#### Nanopore library preparation and sequencing

Genomic DNA extracted from *Pseudomonas aeruginosa* strain KAEH25 (≥ 50 ng µL⁻^1^; A₂₆₀/₂₈₀ ≈ 1.8–2.0) was used to prepare an Oxford Nanopore Technologies (ONT) sequencing library with the Ligation Sequencing Kit (SQK-LSK109, ONT). End-repair and dA-tailing were performed using the NEBNext® Ultra™ II End Repair/dA-Tailing Module (New England BioLabs, Cat. No. E7546), followed by adapter ligation with the NEBNext Quick T4 DNA Ligase (NEB, M2200) and ONT-supplied adapters. Approximately 400 ng of the final library was mixed with ONT Library Loading Beads (EXP-LLB001) and loaded onto a primed R10.4.1 flow cell (FLO-MIN106D) installed in a MinION Mk1C device. Sequencing was carried out for 48 h with continuous acquisition using MinKNOW Core v21.02.3. Base-calling was performed in real time with Guppy v6.4.6 (high-accuracy model). Resulting FASTQ files were imported into Geneious Prime® 2025.0.5 for adapter trimming and quality filtering (Q ≥ 12). De novo assembly was conducted using Flye v2.9.2 (repeat-graph mode, –nano-corr), and the draft assembly was polished with Medaka v1.7.2 to generate a high-contiguity genome suitable for downstream analyses.

### Genome assembly and annotation

Raw Nanopore sequencing reads were imported into Geneious Prime® 2025.0.5 (Biomatters Ltd., Auckland, New Zealand) for quality control, base-calling verification, and assembly. Adapter and low-quality regions were trimmed using the Geneious built-in trimming tool (minimum quality threshold Q ≥ 12). De novo assembly was performed using the Flye v2.9.2 plugin with default parameters for long-read assemblies. Polishing of the draft genome was conducted using the Medaka v1.7.2 plugin integrated within Geneious Prime. Open reading frames (ORFs) were predicted using the Prodigal plugin (default bacterial gene-calling settings). Functional annotation of predicted ORFs was conducted through BLASTx searches against the NCBI non-redundant (nr) protein database. Additional annotation, gene clustering, and biosynthetic-locus characterization were performed using the Bacterial and Viral Bioinformatics Resource Center (BV-BRC) online platform, which provided automated annotation workflows and subsystem-based gene classification.

### InterProScan & secondary-structure prediction

Amino-acid sequences of phenazine-biosynthetic enzymes were analyzed using multiple bioinformatic tools. Domain identification was performed using the Pfam database, and functional annotations were cross-validated with Gene3D, Panther, and PRINTS. Secondary-structure elements were predicted from sequence alignments using standard structural prediction tools integrated within each platform. Three-dimensional protein structures were generated using AlphaFold, and predicted models were processed and visualized in PyMOL. Conserved residues were examined to locate putative active sites and binding regions. To assess structural correspondence, all predicted models were compared against experimentally resolved protein structures obtained from the Protein Data Bank (PDB).

### Culture preparation and 1‑OH‑PHZ production

A culture of *P. aeruginosa* was initiated by transferring a single colony from a Nutrient Agar (NA; HiMedia, India) plate into 50 mL King’s A medium (HiMedia, India) contained in a 250 mL Erlenmeyer flask. The medium was prepared at pH 7.2 and contained 20 g L⁻^1^ proteose peptone, 10 g L⁻^1^ glycerol, 1.4 g L⁻^1^ MgCl₂·6H₂O, and 10 g L⁻^1^ K₂SO₄. Cultures were incubated at 37 °C with orbital shaking at 180 rpm for 48–72 h using a New Brunswick Scientific incubator to ensure adequate aeration for pigment production.

### 1‑OH‑PHZ extraction

Following incubation, cultures were centrifuged at 10,000 × g for 10 min at 4 °C using a high-speed refrigerated centrifuge (Eppendorf, Germany) to pellet the cells. The resulting supernatant was acidified to pH 2.0 with 1 N HCl (Merck, Germany) and extracted with an equal volume of ethyl acetate (Sigma-Aldrich, USA) in a separatory funnel. The extraction step was repeated three times. The combined organic phases were dried over anhydrous Na₂SO₄ (Thermo Fisher Scientific, USA) and concentrated under reduced pressure at 40 °C using a rotary evaporator (Buchi, Switzerland). The dried extract was resuspended in HPLC-grade methanol (Merck, Germany) for subsequent analysis.

### Quantification of 1‑OH‑PHZ by UV–Vis Spectrophotometry

Quantification of 1‑OH‑PHZ was performed using UV–Vis spectrophotometry at 261 nm with a Shimadzu UV-2600 spectrophotometer equipped with 1 cm quartz cuvettes [37]. A stock solution (1 mg mL⁻^1^) was prepared in 5% (v/v) DMSO and serially diluted to obtain calibration standards ranging from 0–20 µg mL⁻^1^. Test samples were diluted as required to ensure absorbance values remained within the linear range of 0.1–1.0 AU. All measurements were blank-corrected using 5% DMSO, and absorbance was recorded in triplicate. A calibration curve was constructed by linear regression using the equation A = (0.130 ± 0.005) c + (0.002 ± 0.001) (R^2^ ≥ 0.999), where *A* is absorbance and *c* is concentration (µg mL⁻^1^). Sample concentrations were calculated using cₛₐₘₚₗₑ = (Aₛₐₘₚₗₑ − b)/m × DF, where *m* is the slope (0.130 mL µg⁻^1^ cm⁻^1^), *b* is the intercept (0.002), and *DF* is the dilution factor. Samples with absorbance > 1.0 AU were diluted and remeasured. All solutions were protected from light throughout analysis. Method performance was validated by assessing linearity (0–20 µg mL⁻^1^, R^2^ ≥ 0.999), accuracy (98.5–101.2% recovery), precision (RSD ≤ 2% for intra-day and inter-day measurements), and limits of detection and quantification (LOD = 0.15 µg mL⁻^1^; LOQ = 0.50 µg mL⁻^1^). Absorbance values were processed using SpectraGryph 1.2 software, and final concentrations were calculated accordingly.

### Experimental design

Statistical optimization was performed using a sequential two-stage design-of-experiments (DoE) strategy. In the first stage, a Plackett–Burman design (PBD) was employed as a screening tool to efficiently identify the most influential factors affecting 1-OH-PHZ production from a set of nine independent variables. PBD is a two-level fractional factorial design that estimates main effects with minimal runs, assuming that higher-order interactions are negligible. In the second stage, significant factors identified from PBD (p < 0.05) were further optimized using a Central Composite Design (CCD) under Response Surface Methodology (RSM). CCD enables estimation of quadratic effects, curvature, and interaction terms, thereby allowing identification of the true optimal conditions within the experimental domain. This sequential approach, screening followed by optimization, adheres to established DoE best practices and avoids the inefficiency of directly applying RSM to all nine variables.

### Plackett–Burman design (PBD) for screening of factors influencing 1‑OH‑PHZ production

PBD was used to screen nine independent variables influencing 1‑OH‑PHZ production. Each factor was evaluated at two levels, coded as low (–1) and high (+ 1). The variables included temperature (30–37 °C), pH (6.0–7.0), glucose concentration (1–2%), peptone concentration (1–2 g L⁻^1^), inoculum size (5–10 mL), incubation time (24–48 h), agitation speed (100–200 rpm), iron concentration (0.1–0.2 mM), and phosphate buffer concentration (0–10 mM). Experimental runs were generated according to the PBD matrix, and each condition was tested in triplicate. The measured response was the concentration of 1‑OH‑PHZ produced under each set of conditions (Table 1).

**Table 1:** Plackett–Burman design matrix for screening 9 factors affecting 1‑OH‑PHZ production

Factor	Name	Units	Low Level (− 1)	High Level (+ 1)
A	Temperature	(°C)	30.00	37.00
B	pH		6.00	7.00
C	Glucose	(%)	1.0000	2.00
D	peptone	g/L	1.0000	2.00
E	Inoculum Size	mL	5.00	10.00
F	Incubation Time	h	24.00	48.00
G	Agitation Speed	rpm	100.00	200.00
H	Iron Concentration	mM	0.1000	0.2000
J	Phosphate Buffer	mM	0.0000*	10.00

^*^0.0000 mM indicates no added phosphate buffer to the base medium

### Experimental procedure

The PBD matrix was generated using Design-Expert v13.0, resulting in 12 experimental runs. For each run, the production medium was prepared according to the factor levels specified in the design matrix. Inoculum was prepared by incubating the bacterial strain in seed culture medium for 18 h at 30 °C and 150 rpm. Fermentation was conducted in 250 mL Erlenmeyer flasks containing 50 mL of production medium, incubated under the conditions assigned for each experimental run. All experiments were performed in triplicate.

### Analytical methods

Following incubation, culture broths were centrifuged at 10,000 × g for 15 min at 4 °C. The supernatant was collected, and 1‑OH‑PHZ was quantified by UV detection at 261 nm. Concentrations were determined by comparing absorbance-derived peak areas with those of authentic standards using SpectraGryph 1.2 software, and values were converted to corresponding concentrations using the established calibration curve.

### Statistical analysis

Data from the Plackett–Burman experiments were analyzed by multiple linear regression using a first-order model on coded variables:

$Y={\beta}_{0}+\sum {\beta}_{i}{X}_{i}$where $Y$ is 1‑OH‑PHZ concentration, ${\beta}_{0}$ is the intercept, ${\beta}_{i}$ are regression coefficients, and ${X}_{i}$ are coded factor levels (− 1, + 1). Coefficients were estimated by least squares in Design-Expert v13.0. Factor significance was assessed with two-sided Student’s *t*-tests at $\alpha =0.05$. Model adequacy was evaluated by analysis of variance (ANOVA), including ${R}^{2}$ and adjusted ${R}^{2}$. Standardized effect estimates were visualized using Pareto charts. Factors with $p<0.05$ were advanced to subsequent response-surface optimization.

### Central-composite design (CCD)

In accordance with the statistical principles articulated by Asitok et al. (2022), it was specified that RSM using a Central Composite Design (CCD) was applied only after significant factors had been identified through the PBD, thereby maintaining the sequential and hierarchical structure required for design-of-experiments methodology [38]. A CCD was employed to optimize the variables influencing 1‑OH‑PHZ production. The design incorporated factorial points, axial points, and replicated center points to enable estimation of curvature and fitting of a second-order polynomial model.

For the Central Composite Design (CCD), each factor was evaluated at five coded levels: − α (axial low), − 1 (factorial low), 0 (center), + 1 (factorial high), and + α (axial high). The axial distance α was calculated based on rotatability using the equation $\alpha ={\left({2}^{k}\right)}^\frac{1}{4}$, where k is the number of factorial points in the fractional design. For this Min-Run Resolution V design, the software computed α = 1.682. The axial points extend beyond the factorial region to allow accurate estimation of quadratic curvature. Table 2 summarizes the coded levels and their corresponding actual experimental values. In total, eight variables were investigated across these five coded levels: temperature (A), pH (B), glucose concentration (C), peptone concentration (D), inoculum size (E), incubation time (F), agitation speed (G), and phosphate buffer concentration (H). Iron concentration was fixed at 0.15 mM based on its minimal effect in the screening phase. The design comprised 54 non-center points and 6 center points, giving a total of 60 experimental runs.

**Table 2:** Central Composite Design (CCD) parameters for 1-OH-PHZ optimization showing actual factor ranges, axial distances, and coded levels

Factor	Name	Units	–α (axial low)	–1 (factorial low)	0 (center)	+ 1 (factorial high)	+ α (axial high)
A	Temperature	°C	27.61	30	33.5	37	39.39
B	pH	–	5.66	6	6.5	7	7.34
C	Glucose	%	0.66	1	1.5	2	2.34
D	Peptone	g/L	0.66	1	1.5	2	2.34
E	Inoculum size	mL	3.30	5	7.5	10	11.70
F	Incubation time	h	15.82	24	36	48	56.18
G	Agitation speed	rpm	65.91	100	150	200	234.09
H	Phosphate buffer	mM	–3.41*	0	5	10	13.41

^*^Negative axial value for phosphate buffer indicates that the design space extends below zero, which was treated as 0 mM in actual experiments

The experimental matrix was generated using Design-Expert v13.0. Fermentations were carried out in 250 mL Erlenmeyer flasks containing 50 mL of production medium prepared according to the designated factor levels. Cultures were incubated under the temperature, agitation, and time conditions specified by the CCD matrix. After incubation, all culture samples were harvested, and 1‑OH‑PHZ concentrations were quantified at 261 nm following the previously established spectrophotometric protocol. All experiments were performed in triplicate. Model fitting, ANOVA, diagnostic evaluation, and response-surface generation were conducted using Design-Expert v13.0. The CCD model was assessed for overall significance, lack-of-fit, regression coefficients, and goodness-of-fit statistics including R^2^, adjusted R^2^, and adequate precision.

### Antimicrobial activity

Antimicrobial susceptibility testing was carried out following CLSI (2024) guidelines to provide preliminary, proof-of-concept confirmation of the antibacterial activity of 1-OH-PHZ. Test organisms (*S. enterica* serovar Typhimurium ATCC 14028, *S. aureus* ATCC 25923, *E. coli* ATCC 25922, *K. pneumoniae* KCTC 22482, *E. faecalis* ATCC 29212, and *L. monocytogenes* ATCC 19115) were revived on 5% sheep-blood agar and incubated at 37 ± 1 °C for 18–24 h. Single colonies were transferred to cation-adjusted Mueller–Hinton broth and grown to late exponential phase at 37 ± 1 °C and 150 rpm. Cell suspensions were standardized to 0.5 McFarland (~ 1.5 × 10⁸ CFU mL⁻^1^) before streaking onto Mueller–Hinton agar plates. Wells (6 mm) were filled with 50 µL of 1-OH-PHZ (1 mg mL⁻^1^ in 5% DMSO; final 20 µg/well). Antibiotic controls included ciprofloxacin (5 µg), tetracycline (30 µg), and ampicillin (10 µg). Plates were incubated at 37 ± 1 °C for 18 h, and inhibition zones were recorded in triplicate. As this study focused on verifying bioactivity rather than establishing antimicrobial potency, minimum inhibitory concentration (MIC) assays were not performed here; however, MIC determinations for 1-OH-PHZ have been reported in a separate study and will complement future investigations [39–41].

## Results

### 16S rRNA phylogenetic identification

**Fig. 1:**
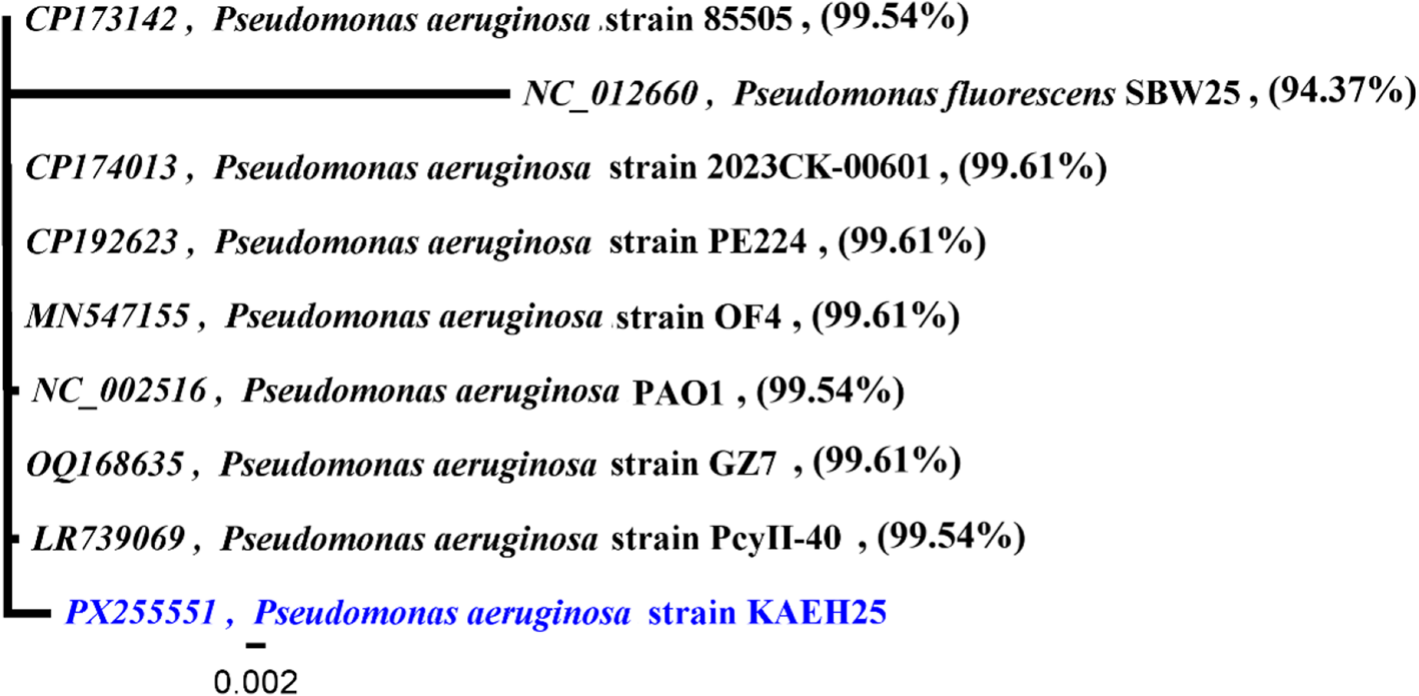
Maximum-likelihood phylogeny of *P. aeruginosa* strain KAEH25

Amplification of the 16S rRNA gene from *P. aeruginosa* strain KAEH25 yielded a contiguous 1,524-bp sequence deposited in GenBank under accession number PX255551. BLASTn analysis showed ≥ 99.54% nucleotide identity to all *P. aeruginosa* reference and clinical strains, with four strains—CP174013, CP192623, MN547155, and OQ168635—sharing 99.61% identity. The closest non-*aeruginosa* match, *P. fluorescens* SBW25, exhibited 94.37% identity (1,416/1,502 bp). Maximum-likelihood phylogenetic reconstruction placed strain KAEH25 within a strongly supported *P. aeruginosa* clade (bootstrap 100%), clearly separated from *P. fluorescens* SBW25 (Fig. 1).

### UV–Vis spectrum of 1‑OH‑PHZ

**Fig. 2:**
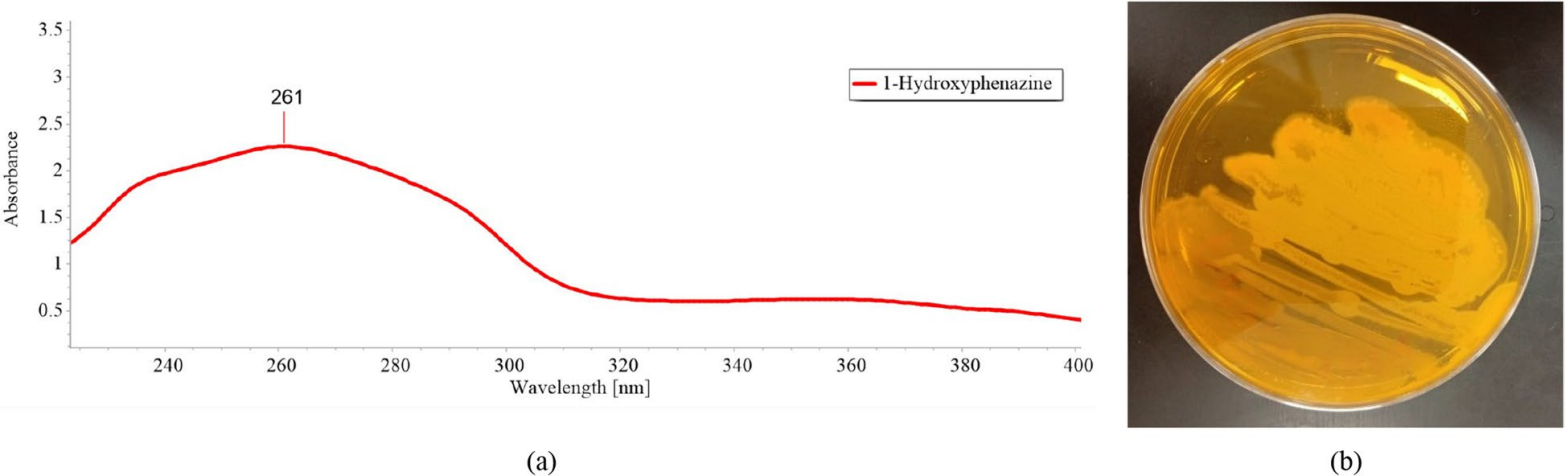
Spectral and phenotypic characteristics of 1‑OH‑PHZ. **a** UV–Vis spectrum indicating a strong absorption maximum at 261 nm and minor bands at higher wavelengths. **b** Representative plate image showing the characteristic orange pigment

The UV–Vis absorption spectrum of 1‑OH‑PHZ (Fig. 2a) shows a dominant absorption maximum at 261 nm, representing the highest-intensity feature in the spectrum. A shoulder is observed near 240 nm, with additional weak features appearing around 280 nm and within the 300–340 nm region. The strong band at 261 nm (A ≈ 2.6) reflects a π → π* transition associated with the compound’s conjugated aromatic system. Beyond the primary maximum, the spectrum displays a gradual decline in absorbance from 280 to 400 nm, indicating reduced electronic transition probability at longer wavelengths. In parallel, the pigment exhibited its characteristic orange coloration when visualized on agar plates (Fig. 2b), consistent with phenazine production.

### Nanopore sequencing and assembly of the phenazine biosynthetic locus

**Fig. 3:**
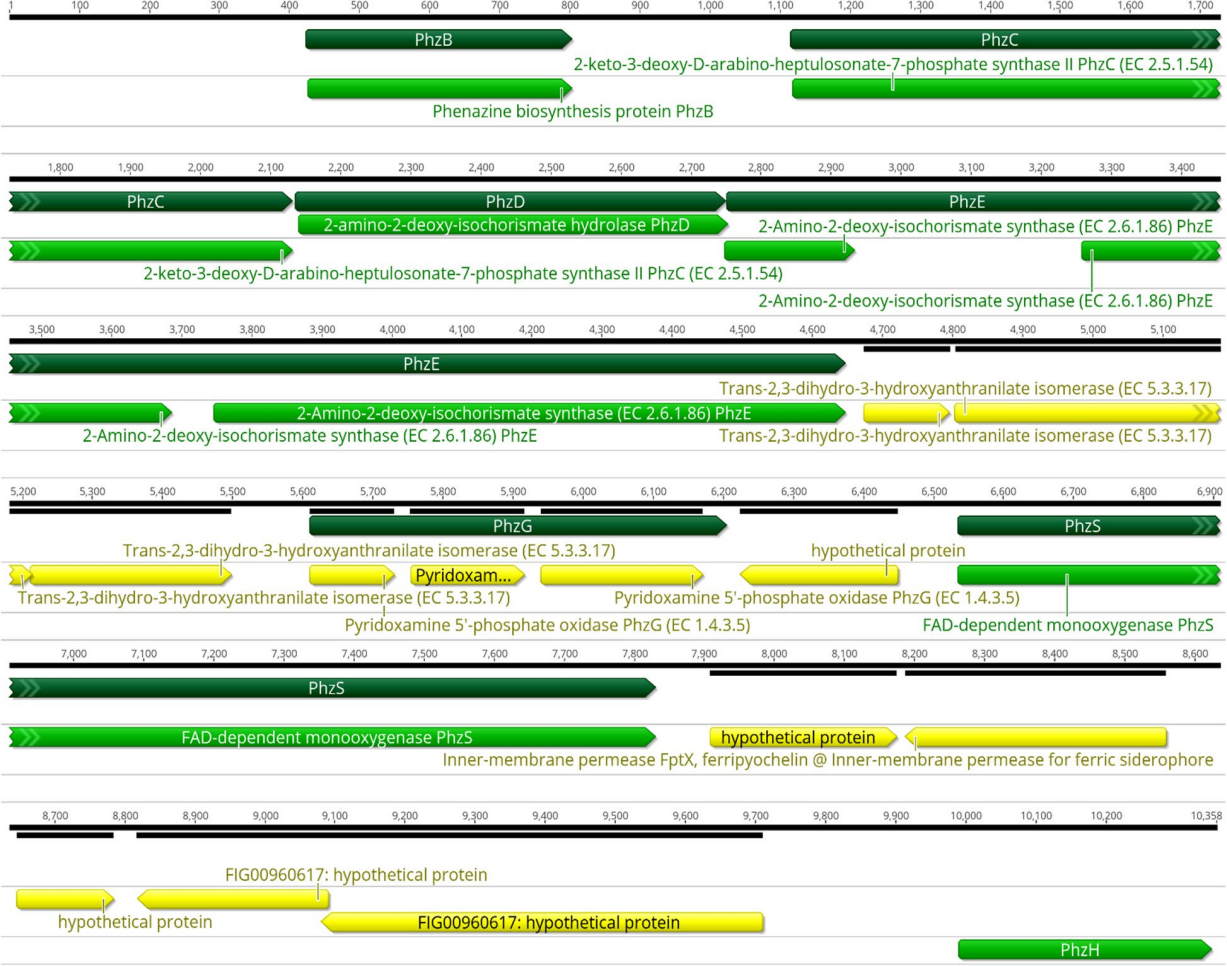
Schematic representation of Nanopore long-read assembly of the 10,457-bp phenazine biosynthetic locus in *P. aeruginosa*

Quality assessment of the Nanopore dataset revealed 1,222 high-quality reads totaling ~ 2.3 Mbp with a GC content of 62%. Read-length distribution was broad, no adapter contamination or overrepresented sequences were detected, and no ambiguous bases (N) were present, indicating a clean dataset suitable for assembly. De novo assembly produced a single, gap-free contig corresponding to the phenazine biosynthetic locus. The assembled region included seven contiguous core biosynthetic genes (*phzB–phzH*) arranged collinearly on the forward strand, along with the upstream *phzF* isomerase gene (Fig. 3). Nanopore sequencing captured the entire locus within a 10,358-bp read, spanning positions 424–10,358. All biosynthetic genes, as well as adjacent flanking genes, were fully resolved, with lengths and orientations summarized in Table 3.

**Table 3:** Gene organization and structural features of the phenazine biosynthetic locus in *P. aeruginosa* KAEH25, including gene coordinates, lengths, orientations, and annotated functions

Element	Coordinates (bp)	Length (bp)	Direction	Function
*phzB*	426–803	378	Forward	Phenazine biosynthesis protein
*phzC*	1 118–2 131	1014	Forward	2-keto-3-deoxy-D-arabino-heptulosonate-7-phosphate synthase
*phzD*	2 140–2 751	612	Forward	2-amino-2-deoxy-isochorismate hydrolase
*phzE*	2 881–4 646	1896	Forward	2-amino-2-deoxy-isochorismate synthase
*phzG*	5 611–6 204	594	Forward	Pyridoxamine 5′-phosphate oxidase
*phzS*	6 535–7 830	1 296	Forward	FAD-dependent mono-oxygenase
*phzH*	9 991–10 350	360	Forward	Phenazine-modifying enzyme
Flanking genes captured in the same read				
Gene	Coordinates (bp)	Length (bp)	Direction	Function
*fptX*	8 187–8 558	372	Reverse	Inner-membrane permease (ferripyochelin uptake)
hypothetical-1	8 647–8 784	138	Forward	Hypothetical protein
hypothetical-2	8 818–9 090	273	Reverse	FIG00960617 hypothetical
hypothetical-3	9 081–9 710	630	Reverse	FIG00960617 hypothetical

### InterProScan annotation and secondary-structure prediction

**Fig. 4:**
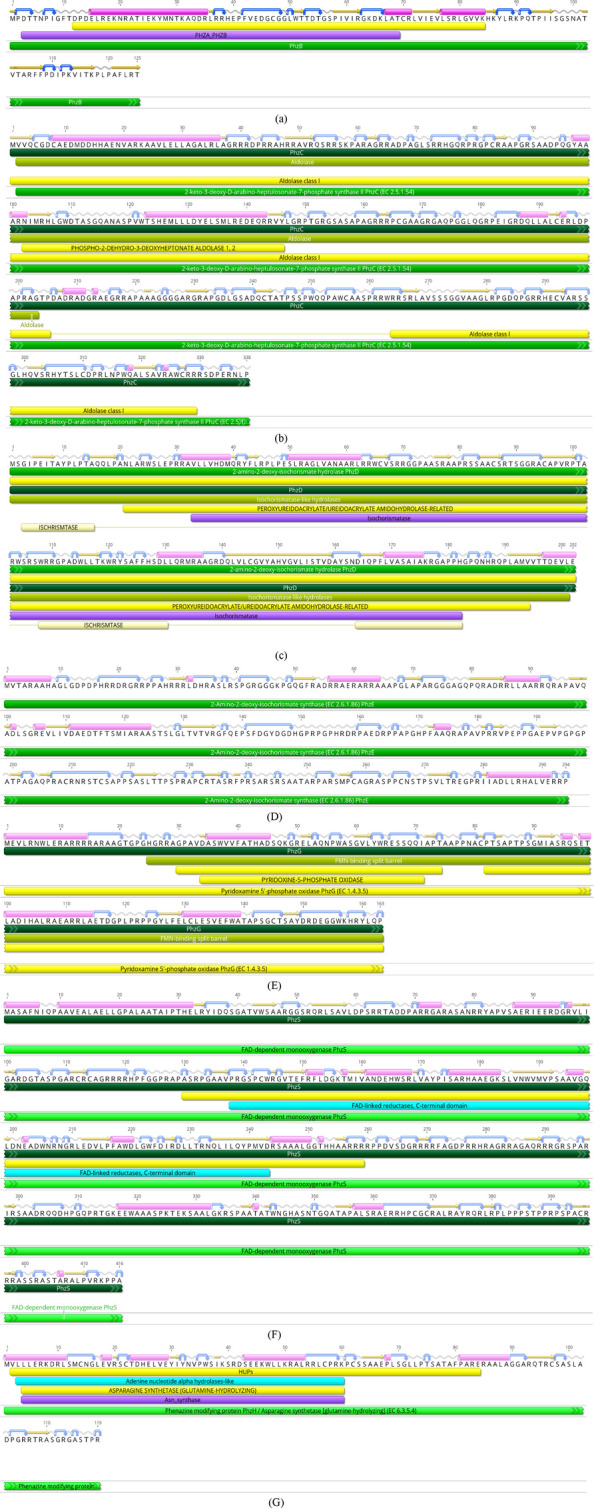
Interproscan and 2ry structure prediction of (**a**) PhzB, **b** PhzC, **c** PhzD, **d** PhzE, **e** PhzG, **f** PhzS, and **g** PhzH protein

InterProScan and secondary-structure analysis identified conserved domains and characteristic folds across all phenazine-biosynthetic enzymes (Fig. 4). PhzB (126 aa) contained a PHZA/PHZB Pfam domain (aa 3–69) and exhibited an N-terminal α-helix followed by a compact β-sandwich (Fig. 4a). PhzC (339 aa) displayed a typical TIM-barrel architecture consistent with 2-keto-3-deoxy-D-arabino-heptulosonate-7-phosphate synthase II, with alternating β-strands and α-helical inserts forming the catalytic barrel (Fig. 4b). PhzD (203 aa) showed an isochorismatase-type fold consisting of a central parallel β-sheet flanked by seven α-helices that form the predicted substrate-binding pocket (Fig. 4c).

**Fig. 5:**
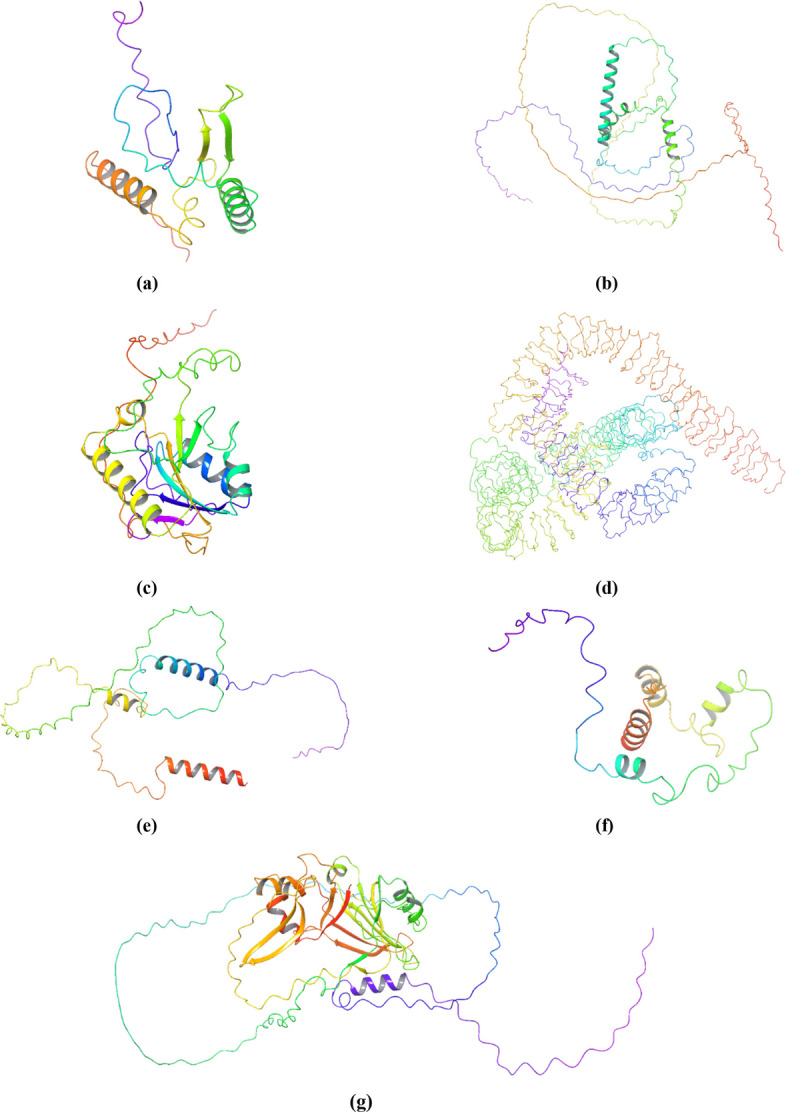
AlphaFold-predicted 3D models of (**a**) PhzB, **b** PhzC, **c** PhzD, **d** PhzE, **e** PhzG, **f** PhzH, and **g** PhzS

The N-terminal region of PhzE (≥ 866 aa) adopted an aminotransferase-like α/β fold characteristic of PLP-dependent enzymes, with multiple β-strands interspersed among α-helices (Fig. 4d). PhzG (322 aa) demonstrated an FMN-binding split-barrel domain architecture, featuring a curved β-sheet surrounding a helical core typical of pyridoxamine-5′-phosphate oxidases (Fig. 4e). PhzS (417 aa) possessed an FAD-dependent monooxygenase fold, including an N-terminal helical cap and an extended Rossmann-like β-sheet associated with FAD/NAD(P)H interaction (Fig. 4f). PhzH (120 aa) showed a HUP-family nucleotide-binding fold comprising an N-terminal helix, a βαβ motif, and several helical inserts consistent with glutamine-dependent amidase activity (Fig. 4g). AlphaFold-based modeling produced high-confidence three-dimensional structures for all seven phenazine-biosynthetic enzymes (Fig. 5). These models collectively confirmed that KAEH25 encodes a structurally intact and functionally coherent phenazine biosynthetic pathway.

### Phenazine biosynthetic pathway reconstruction based on gene cluster analysis

**Fig. 6:**
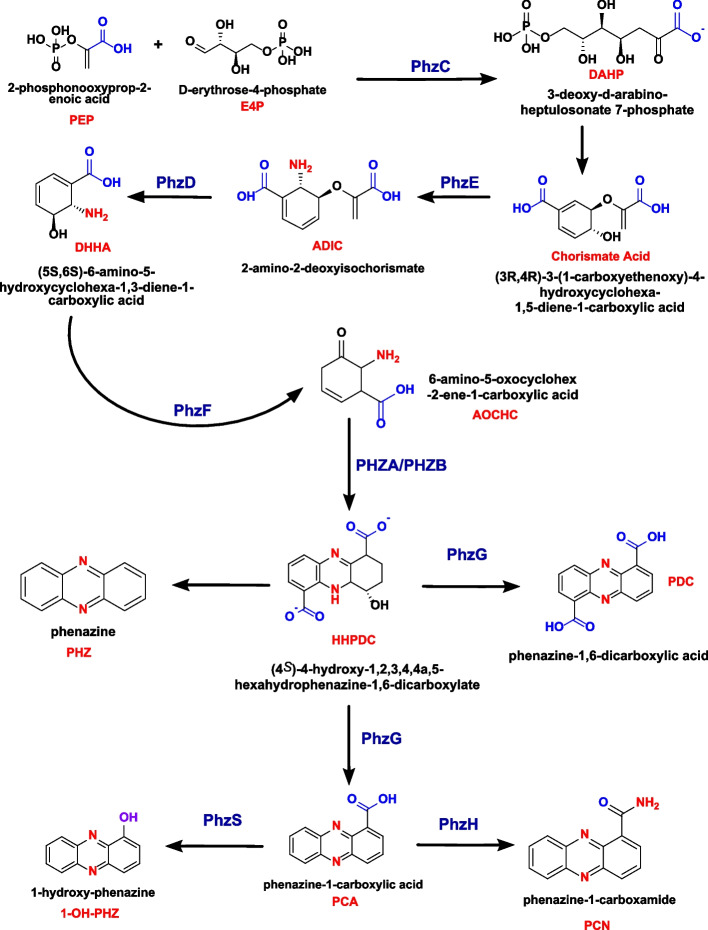
Biosynthetic pathway for phenazine production by *P. aeruginosa* KAEH25* (PX255551)*

Analysis of the assembled locus confirmed the presence and organization of all enzymes required for phenazine biosynthesis in *P. aeruginosa* KAEH25 (Fig. 6). The contig contained the complete set of core biosynthetic genes, including phzE, phzD, phzF, phzB, and phzG, as well as the terminal tailoring genes phzH and phzS, each positioned sequentially along the forward strand. The recovered gene order and orientation corresponded precisely to the canonical phenazine biosynthetic pathway, with the arrangement enabling the stepwise conversion of chorismate-derived intermediates into phenazine-1-carboxylic acid (PCA) and its downstream products. The presence of phzH and phzS further confirmed the pathway’s capacity to direct PCA toward the formation of phenazine-1-carboxamide and 1-OH-PHZ, respectively.

### Plackett–Burman design for optimization of 1‑OH‑PHZ production

Plackett–Burman screening identified substantial variation in 1‑OH‑PHZ production across the 12 experimental runs, with the highest titre (20.9 µg mL⁻^1^) observed in run 8. This condition incorporated a temperature of 37 °C, pH 7, 1% glucose, 2 g L⁻^1^ peptone, a 10% inoculum, 48 h incubation, 100 rpm agitation, 0.1 mM Fe^2^⁺, and no added phosphate buffer. Temperature, peptone concentration, and glucose level showed the most pronounced effects on production. Higher peptone (2 g L⁻^1^) consistently corresponded with increased titres, while 1% glucose supported higher yields than 2%. The absence of phosphate buffer was associated with elevated production in the highest-yielding runs. Operational factors also contributed: agitation at 100 rpm produced higher yields than 200 rpm, and 48 h incubation improved titres relative to 24 h. Inoculum volume exhibited no consistent influence within the tested range. Collectively, the screening data delineated an optimal parameter, 37 °C, pH 7, 1% glucose, 2 g L⁻^1^ peptone, 10% inoculum, 48 h, 100 rpm, 0.1 mM Fe^2^⁺, and zero phosphate buffer, that yielded the highest measured 1‑OH‑PHZ concentration (Table 4).

**Table 4:** Plackett–Burman screening design and optimal conditions for 1‑OH‑PHZ production

Run	A: Temperature	B: pH	C: Glucose	D: peptone	E: Inoculum Size	F: Incubation Time	G: Agitation Speed	H: Iron Concentration	J: Phosphate Buffer	1-Hydroxyphenazine Production
	(°C)		(%)	g/L	mL	h	rpm	mM	mM	µg/ml
1	30	6	2	1	10	48	100	0.2	10	15.2
2	37	7	2	1	5	24	200	0.1	10	9.8
3	30	7	2	2	5	24	100	0.2	0	18.6
4	30	6	1	2	5	48	200	0.1	10	12.4
5	37	7	1	1	5	48	100	0.2	10	11.1
6	37	6	2	2	5	48	200	0.2	0	14.3
7	37	6	1	1	10	24	200	0.2	0	7.5
8	37	7	1	2	10	48	100	0.1	0	20.9
9	30	6	1	1	5	24	100	0.1	0	6.3
10	37	6	2	2	10	24	100	0.1	10	16.7
11	30	7	2	1	10	48	200	0.1	0	19.4
12	30	7	1	2	10	24	200	0.2	10	17.8

**Fig. 7:**
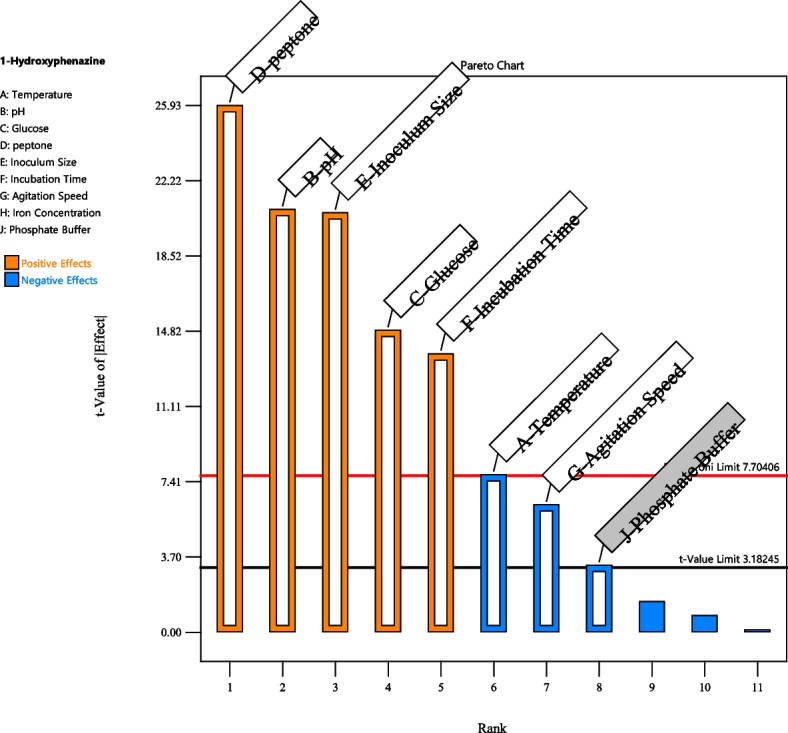
Pareto chart showing the standardized effects of the nine Plackett–Burman factors on 1‑OH‑PHZ production

Based on the Pareto chart, factor significance was evaluated using both the Bonferroni limit (7.70406) and the standard *t*-value limit (3.18245). Factors with *t*-values exceeding the Bonferroni threshold were classified as highly significant contributors to 1-hydroxyphenazine production. These included peptone, pH, inoculum size, glucose concentration, incubation time, and temperature, each demonstrating strong and positive effects. Factors with *t*-values above the standard significance limit but below the Bonferroni threshold—agitation speed, iron concentration, and phosphate buffer—were considered moderately significant, exerting weaker but detectable influences on the response (Fig. 7).

The ANOVA results for the selected factorial model demonstrated that the model was highly significant, with a Model F-value of 256.34, indicating that the likelihood of such a large F-statistic arising from random variation is only 0.04%. All evaluated main factors—temperature (A), pH (B), glucose concentration (C), peptone concentration (D), inoculum size (E), incubation time (F), agitation speed (G), and phosphate buffer (J)—showed *p*-values below 0.05, confirming their significant contributions to 1-hydroxyphenazine production. Among these, peptone (D), pH (B), and inoculum size (E) exhibited the strongest effects, reflected by their high F-values of 672.25, 432.98, and 426.14, respectively. The relatively small residual error (0.3667) further supports the robustness of the model. These findings indicate that all tested factors play meaningful roles in influencing pigment yield, and although model reduction is possible, it should be carried out cautiously to preserve hierarchical integrity (Table 5).

**Table 5:** ANOVA results for selected factorial model of 1‑OH‑PHZ Production

Model	250.64	8	31.33	256.34	0.0004	significant
A-Temperature	7.36	1	7.36	60.25	0.0044	significant
B-pH	52.92	1	52.92	432.98	0.0002	significant
C-Glucose	27.00	1	27.00	220.91	0.0007	significant
D-peptone	82.16	1	82.16	672.25	0.0001	significant
E-Inoculum Size	52.08	1	52.08	426.14	0.0002	significant
F-Incubation Time	22.96	1	22.96	187.88	0.0008	significant
G-Agitation Speed	4.81	1	4.81	39.38	0.0082	significant
J-Phosphate Buffer	1.33	1	1.33	10.91	0.0456	significant
Residual	0.3667	3	0.1222			
Cor Total	251.01	11				

The Predicted R^2^ of 0.9766 is in reasonable agreement with the Adjusted R^2^ of 0.9946; i.e. the difference is less than 0.2. Adeq Precision measures the signal to noise ratio. A ratio greater than 4 is desirable. Your ratio of 48.883 indicates an adequate signal. This model can be used to navigate the design space (Table 6).

**Table 6:** Fit statistics for the PB model

Std. Dev	0.3496	R^2^	0.9985
Mean	14.17	**Adjusted R**^**2**^	0.9946
C.V. %	2.47	**Predicted R**^**2**^	0.9766
		**Adeq Precision**	48.8828

The final regression equation expressed in actual factor units provides a means of predicting 1‑OH‑PHZ concentrations at specified experimental settings. Because the coefficients are scaled to accommodate the original units of each variable and the intercept does not correspond to the design-space center, this equation is intended solely for prediction and not for assessing relative factor importance.

### Regression equation for 1‑OH‑PHZ (in actual factor units)

$$\begin{array}{c}(1-{\boldsymbol{H}}{\boldsymbol{y}}{\boldsymbol{d}}{\boldsymbol{r}}{\boldsymbol{o}}{\boldsymbol{x}}{\boldsymbol{y}}{\boldsymbol{p}}{\boldsymbol{h}}{\boldsymbol{e}}{\boldsymbol{n}}{\boldsymbol{a}}{\boldsymbol{z}}{\boldsymbol{i}}{\boldsymbol{n}}{\boldsymbol{e}}\\=-26.15238-0.22381({\boldsymbol{T}}{\boldsymbol{e}}{\boldsymbol{m}}{\boldsymbol{p}}{\boldsymbol{e}}{\boldsymbol{r}}{\boldsymbol{a}}{\boldsymbol{t}}{\boldsymbol{u}}{\boldsymbol{r}}{\boldsymbol{e}})\\+4.20({\boldsymbol{p}}{\boldsymbol{H}})+3.00({\boldsymbol{G}}{\boldsymbol{l}}{\boldsymbol{u}}{\boldsymbol{c}}{\boldsymbol{o}}{\boldsymbol{s}}{\boldsymbol{e}})+5.23333({\boldsymbol{P}}{\boldsymbol{e}}{\boldsymbol{p}}{\boldsymbol{t}}{\boldsymbol{o}}{\boldsymbol{n}}{\boldsymbol{e}})\\*+0.83333({\boldsymbol{I}}{\boldsymbol{n}}{\boldsymbol{o}}{\boldsymbol{c}}{\boldsymbol{u}}{\boldsymbol{l}}{\boldsymbol{u}}{\boldsymbol{m}}{\boldsymbol{S}}{\boldsymbol{i}}{\boldsymbol{z}}{\boldsymbol{e}})+0.11528({\boldsymbol{I}}{\boldsymbol{n}}{\boldsymbol{c}}{\boldsymbol{u}}{\boldsymbol{b}}{\boldsymbol{a}}{\boldsymbol{t}}{\boldsymbol{i}}{\boldsymbol{o}}{\boldsymbol{n}}{\boldsymbol{T}}{\boldsymbol{i}}{\boldsymbol{m}}{\boldsymbol{e}}))\\-0.01267({\boldsymbol{A}}{\boldsymbol{g}}{\boldsymbol{i}}{\boldsymbol{t}}{\boldsymbol{a}}{\boldsymbol{t}}{\boldsymbol{i}}{\boldsymbol{o}}{\boldsymbol{n}}{\boldsymbol{S}}{\boldsymbol{p}}{\boldsymbol{e}}{\boldsymbol{e}}{\boldsymbol{d}}-0.06667({\boldsymbol{P}}{\boldsymbol{h}}{\boldsymbol{o}}{\boldsymbol{s}}{\boldsymbol{p}}{\boldsymbol{h}}{\boldsymbol{a}}{\boldsymbol{t}}{\boldsymbol{e}}{\boldsymbol{B}}{\boldsymbol{u}}{\boldsymbol{f}}{\boldsymbol{f}}{\boldsymbol{e}}{\boldsymbol{r}}))\end{array}$$

**Fig. 8:**
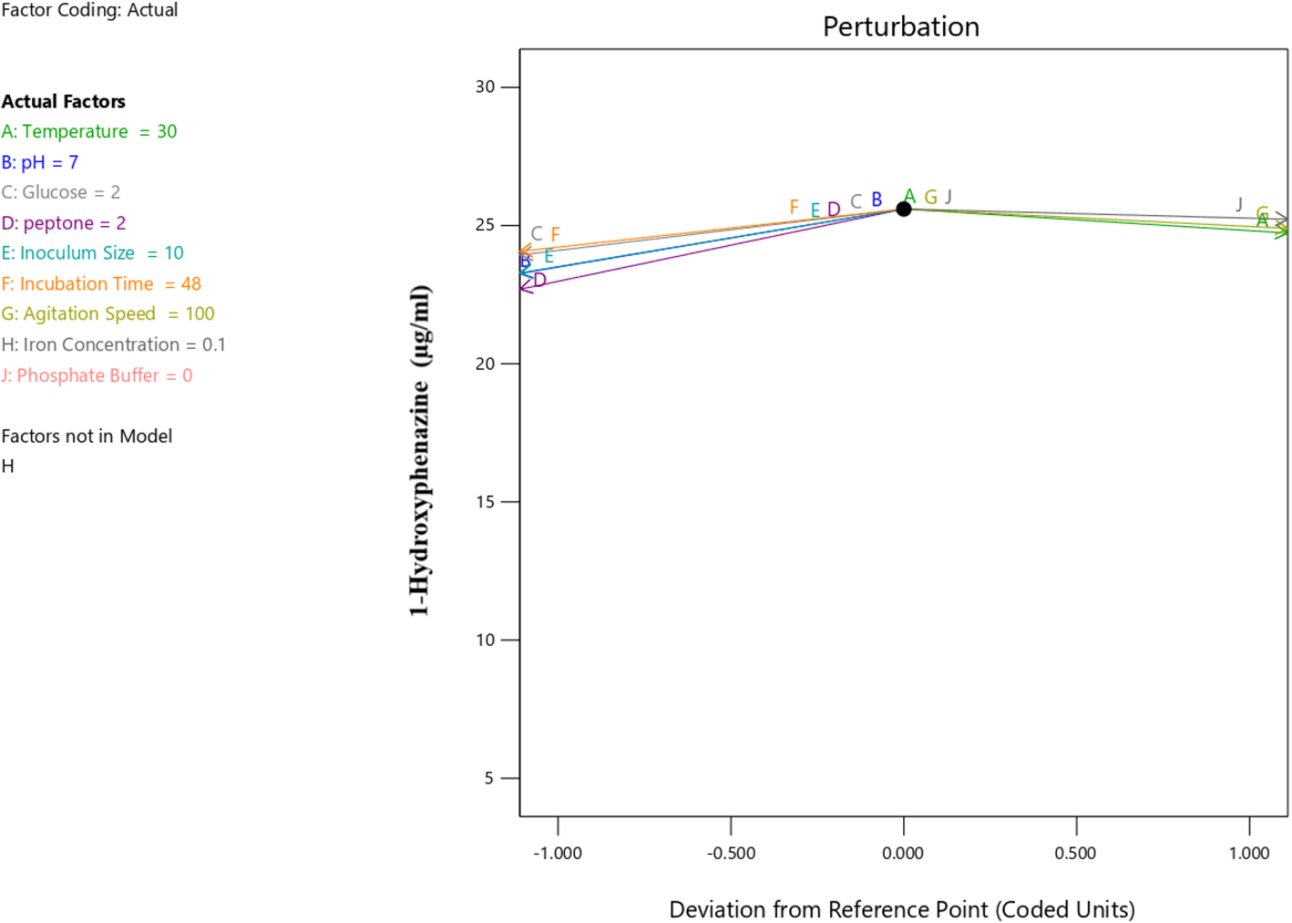
Perturbation plot illustrating the sensitivity of 1‑OH‑PHZ production to individual experimental factors

The perturbation plot (Fig. 8) shows the relative sensitivity of 1‑OH‑PHZ production to changes in each experimental factor. Using the reference point where all variables were set at their actual levels, deviations in either direction generally resulted in reduced production. Among the tested factors, pH (B), peptone concentration (D), and inoculum size (E) produced the steepest response curves, indicating the highest sensitivity to variation from their respective reference levels. Changes in temperature (A), glucose concentration (C), incubation time (F), agitation speed (G), iron concentration (H), and phosphate buffer (J) had comparatively smaller effects on the response, reflecting lower sensitivity within the tested ranges.

### Central-composite design (CCD) and response surface methodology (RSM)

The CCD generated a comprehensive matrix of 60 experimental runs that evaluated the combined effects of nine independent variables—temperature, pH, glucose concentration, peptone concentration, inoculum size, incubation time, agitation speed, iron concentration, and phosphate buffer—on 1-hydroxyphenazine production. The experimental runs captured a wide range of factor combinations, enabling assessment of both linear and nonlinear responses across the design space. Measured 1-hydroxyphenazine concentrations ranged from a minimum of 5.44 µg/mL (Run 12) to a maximum of 24.85 µg/mL (Run 47), demonstrating substantial variability in pigment output under different cultivation conditions. Higher yields were generally associated with runs incorporating near-neutral pH (≈6.5–7.0), moderate glucose levels (≈1–2%), elevated peptone concentrations, larger inoculum volumes, and extended incubation periods. In contrast, low pH, reduced inoculum size, or suboptimal nutrient levels corresponded with markedly lower production. The dataset provides a robust foundation for model fitting and response-surface analysis, enabling identification of significant factors and their interactions that govern optimal 1-hydroxyphenazine biosynthesis (Table 7).

**Table 7:** Central Composite Design (CCD) experimental runs presenting factor combinations and measured 1‑hydroxyphenazine concentrations for all 60 optimization trials

Run	A: Temperature	B: pH	C: Glucose	D: peptone	E: Inoculum Size	F: Incubation Time	G: Agitation Speed	H: Phosphate Buffer	1-Hydroxyphenazine
	**(°C)**		**(%)**	**g/L**	**mL**	**h**	**rpm**	**mM**	**µg/ml**
**1**	**39.3863**	**6.5**	**1.5**	**1.5**	**7.5**	**36**	**150**	**5**	**15.71**
**2**	**37**	**7**	**2**	**1**	**5**	**24**	**100**	**0**	**14.98**
**3**	**33.5**	**6.5**	**1.5**	**1.5**	**7.5**	**36**	**150**	**5**	**16.10**
**4**	**37**	**6**	**2**	**2**	**10**	**48**	**100**	**0**	**15.45**
**5**	**33.5**	**6.5**	**1.5**	**2.3409**	**7.5**	**36**	**150**	**5**	**18.00**
**6**	**33.5**	**6.5**	**1.5**	**1.5**	**7.5**	**36**	**150**	**5**	**16.40**
**7**	**33.5**	**6.5**	**1.5**	**0.65910**	**7.5**	**36**	**150**	**5**	**14.32**
**8**	**37**	**6**	**1**	**1**	**5**	**24**	**100**	**0**	**8.49**
**9**	**33.5**	**6.5**	**1.5**	**1.5**	**7.5**	**15.8185**	**150**	**5**	**11.85**
**10**	**30**	**6**	**1**	**1**	**5**	**48**	**100**	**10**	**14.85**
**11**	**37**	**6**	**1**	**1**	**10**	**24**	**200**	**10**	**6.80**
**12**	**30**	**7**	**1**	**1**	**5**	**24**	**200**	**0**	**5.44**
**13**	**37**	**6**	**2**	**1**	**5**	**48**	**200**	**0**	**17.20**
**14**	**30**	**7**	**1**	**2**	**5**	**48**	**100**	**10**	**18.25**
**15**	**33.5**	**6.5**	**1.5**	**1.5**	**7.5**	**36**	**65.9104**	**5**	**15.81**
**16**	**27.6137**	**6.5**	**1.5**	**1.5**	**7.5**	**36**	**150**	**5**	**16.49**
**17**	**37**	**6**	**1**	**1**	**10**	**48**	**100**	**10**	**17.56**
**18**	**30**	**6**	**2**	**2**	**10**	**24**	**200**	**0**	**15.74**
**19**	**30**	**7**	**1**	**2**	**10**	**48**	**200**	**0**	**22.85**
**20**	**33.5**	**6.5**	**1.5**	**1.5**	**7.5**	**36**	**150**	**5**	**16.26**
**21**	**33.5**	**6.5**	**1.5**	**1.5**	**3.29552**	**36**	**150**	**5**	**11.95**
**22**	**33.5**	**6.5**	**0.65910**	**1.5**	**7.5**	**36**	**150**	**5**	**12.12**
**23**	**30**	**7**	**2**	**1**	**10**	**48**	**200**	**0**	**18.88**
**24**	**30**	**6**	**1**	**2**	**5**	**24**	**200**	**10**	**9.85**
**25**	**37**	**7**	**2**	**1**	**10**	**24**	**200**	**10**	**19.69**
**26**	**37**	**7**	**1**	**2**	**10**	**24**	**200**	**10**	**21.40**
**27**	**37**	**6**	**1**	**2**	**5**	**48**	**200**	**0**	**10.46**
**28**	**37**	**7**	**2**	**2**	**5**	**48**	**200**	**0**	**20.16**
**29**	**30**	**7**	**2**	**2**	**10**	**24**	**100**	**0**	**23.15**
**30**	**30**	**6**	**2**	**1**	**5**	**24**	**200**	**10**	**18.06**
**31**	**37**	**7**	**1**	**1**	**5**	**48**	**200**	**10**	**14.96**
**32**	**33.5**	**6.5**	**1.5**	**1.5**	**7.5**	**36**	**150**	**5**	**15.83**
**33**	**37**	**6**	**2**	**2**	**5**	**48**	**100**	**10**	**10.42**
**34**	**30**	**7**	**2**	**2**	**5**	**24**	**200**	**10**	**20.00**
**35**	**37**	**7**	**1**	**2**	**5**	**24**	**100**	**0**	**11.47**
**36**	**33.5**	**6.5**	**2.3409**	**1.5**	**7.5**	**36**	**150**	**5**	**19.42**
**37**	**33.5**	**5.6591**	**1.5**	**1.5**	**7.5**	**36**	**150**	**5**	**10.78**
**38**	**30**	**7**	**1**	**1**	**5**	**24**	**100**	**10**	**5.80**
**39**	**37**	**6**	**2**	**2**	**10**	**24**	**100**	**10**	**15.57**
**40**	**33.5**	**6.5**	**1.5**	**1.5**	**7.5**	**36**	**150**	**13.409**	**15.55**
**41**	**33.5**	**6.5**	**1.5**	**1.5**	**11.7045**	**36**	**150**	**5**	**19.87**
**42**	**33.5**	**6.5**	**1.5**	**1.5**	**7.5**	**36**	**150**	**−3.40896**	**15.80**
**43**	**33.5**	**6.5**	**1.5**	**1.5**	**7.5**	**56.1815**	**150**	**5**	**20.22**
**44**	**30**	**7**	**1**	**1**	**10**	**48**	**200**	**10**	**15.85**
**45**	**33.5**	**6.5**	**1.5**	**1.5**	**7.5**	**36**	**150**	**5**	**15.91**
**46**	**33.5**	**7.3409**	**1.5**	**1.5**	**7.5**	**36**	**150**	**5**	**21.15**
**47**	**37**	**7**	**2**	**2**	**10**	**48**	**100**	**10**	**24.85**
**48**	**37**	**6**	**2**	**2**	**5**	**24**	**200**	**10**	**10.70**
**49**	**30**	**6**	**2**	**1**	**10**	**24**	**100**	**0**	**10.00**
**50**	**37**	**7**	**1**	**1**	**10**	**24**	**100**	**0**	**10.13**
**51**	**33.5**	**6.5**	**1.5**	**1.5**	**7.5**	**36**	**150**	**5**	**15.60**
**52**	**37**	**6**	**2**	**2**	**10**	**48**	**200**	**10**	**15.30**
**53**	**30**	**6**	**2**	**2**	**5**	**24**	**100**	**0**	**16.40**
**54**	**30**	**6**	**2**	**2**	**10**	**48**	**100**	**10**	**21.10**
**55**	**33.5**	**6.5**	**1.5**	**1.5**	**7.5**	**36**	**234.09**	**5**	**17.91**
**56**	**37**	**7**	**1**	**1**	**10**	**48**	**200**	**0**	**15.89**
**57**	**30**	**7**	**2**	**1**	**5**	**48**	**100**	**10**	**19.38**
**58**	**30**	**6**	**1**	**2**	**10**	**24**	**100**	**0**	**18.95**
**59**	**30**	**7**	**1**	**1**	**5**	**48**	**100**	**0**	**6.99**
**60**	**30**	**6**	**1**	**1**	**10**	**48**	**200**	**0**	**17.50**

### ANOVA for two factors in response surface model (2FI)

The ANOVA for the 2FI model demonstrated that the fitted regression was highly significant, with a Model F-value of 15.54, indicating that the probability of such a large F-statistic arising from random experimental variation is extremely small (*p* < 0.0001). Several linear factors exhibited strong and statistically significant effects on 1-hydroxyphenazine production, including pH (B), glucose concentration (C), peptone concentration (D), inoculum size (E), and incubation time (F), each showing high F-values (32.02–51.84) and p-values below 0.0001. Temperature (A) and agitation speed (G) did not contribute significantly within the tested range. Multiple two-factor interactions were also significant, highlighting important synergistic effects among variables. Notable interactions included AB, BC, BD, BH, CD, CE, CF, CG, DE, DG, and EG, all with *p*-values < 0.05, indicating that combinations of pH, glucose, peptone, inoculum size, and incubation time strongly influenced pigment biosynthesis. In contrast, several interaction terms such as AC, AG, AH, BF, CH, DH, and FH were non-significant, suggesting limited or no interactive influence under the evaluated conditions. The model exhibited a significant lack of fit (F = 28.36; *p* = 0.0008), indicating that although the model captures major trends, additional higher-order terms or model refinement may further improve predictive accuracy. Nonetheless, the overall model significance and the strong contribution of key linear and interaction terms confirm that nutrient composition and culture conditions play dominant roles in regulating 1-hydroxyphenazine production (Table 8). The model-fit statistics further describe the performance of the 2FI model. The standard deviation was 1.39, with a mean response of 15.56 µg/mL, yielding a coefficient of variation (C.V.) of 8.95%, which indicates acceptable experimental precision. The model achieved a high R^2^ of 0.9605, demonstrating that 96.05% of the variation in 1-hydroxyphenazine production was explained by the model. However, the Adjusted R^2^ (0.8987) and Predicted R^2^ (–2.3126) were not in agreement, with the negative predicted value indicating that the model performs poorly in predicting new observations and that the overall mean may serve as a better predictor than the current model structure. This discrepancy suggests possible overfitting or the need for higher-order or alternative modeling approaches. Despite this limitation, the Adequate Precision value of 19.1081 greatly exceeds the recommended threshold of 4, confirming a strong signal-to-noise ratio and indicating that the model provides sufficient resolution for navigating the design space.

**Table 8:** ANOVA results for the 2FI model and model coefficients

Model	1085.66	36	30.16	15.54	< 0.0001	significant
A-Temperature	**3.29**	**1**	**3.29**	**1.70**	**0.2057**	non-significant
B-pH	**98.37**	**1**	**98.37**	**50.70**	** < 0.0001**	significant
C-Glucose	**100.59**	**1**	**100.59**	**51.84**	** < 0.0001**	significant
D-peptone	**98.11**	**1**	**98.11**	**50.56**	** < 0.0001**	significant
E-Inoculum Size	**62.13**	**1**	**62.13**	**32.02**	** < 0.0001**	significant
F-Incubation Time	**88.63**	**1**	**88.63**	**45.68**	** < 0.0001**	significant
G-Agitation Speed	**2.76**	**1**	**2.76**	**1.42**	**0.2450**	non-significant
H-Phosphate Buffer	**5.84**	**1**	**5.84**	**3.01**	**0.0962**	non-significant
AB	**27.38**	**1**	**27.38**	**14.11**	**0.0010**	significant
AC	**1.64**	**1**	**1.64**	**0.8474**	**0.3668**	non-significant
AD	**6.88**	**1**	**6.88**	**3.54**	**0.0725**	non-significant
AE	**5.90**	**1**	**5.90**	**3.04**	**0.0946**	non-significant
AF	**7.27**	**1**	**7.27**	**3.75**	**0.0652**	non-significant
AG	**0.2351**	**1**	**0.2351**	**0.1212**	**0.7309**	non-significant
AH	**0.0195**	**1**	**0.0195**	**0.0101**	**0.9210**	non-significant
BC	**25.08**	**1**	**25.08**	**12.93**	**0.0015**	significant
BD	**23.53**	**1**	**23.53**	**12.13**	**0.0020**	significant
BE	**7.91**	**1**	**7.91**	**4.08**	**0.0553**	non-significant
BF	**1.66**	**1**	**1.66**	**0.8576**	**0.3640**	non-significant
BG	**4.95**	**1**	**4.95**	**2.55**	**0.1238**	non-significant
BH	**10.13**	**1**	**10.13**	**5.22**	**0.0319**	significant
CD	**20.76**	**1**	**20.76**	**10.70**	**0.0034**	significant
CE	**30.34**	**1**	**30.34**	**15.63**	**0.0006**	significant
CF	**13.43**	**1**	**13.43**	**6.92**	**0.0149**	significant
CG	**10.23**	**1**	**10.23**	**5.27**	**0.0311**	significant
CH	**0.1518**	**1**	**0.1518**	**0.0783**	**0.7822**	non-significant
DE	**51.01**	**1**	**51.01**	**26.29**	** < 0.0001**	significant
DF	**5.14**	**1**	**5.14**	**2.65**	**0.1173**	non-significant
DG	**10.40**	**1**	**10.40**	**5.36**	**0.0299**	significant
DH	**1.45**	**1**	**1.45**	**0.7455**	**0.3968**	non-significant
EF	**6.81**	**1**	**6.81**	**3.51**	**0.0737**	non-significant
EG	**9.26**	**1**	**9.26**	**4.77**	**0.0394**	significant
EH	**0.0167**	**1**	**0.0167**	**0.0086**	**0.9269**	non-significant
FG	**2.75**	**1**	**2.75**	**1.42**	**0.2456**	non-significant
FH	**2.51**	**1**	**2.51**	**1.30**	**0.2667**	non-significant
GH	**3.45**	**1**	**3.45**	**1.78**	**0.1956**	non-significant
Residual	**44.63**	**23**	**1.94**			
Lack of Fit	**44.19**	**18**	**2.46**	**28.36**	**0.0008**	**significant**
Pure Error	**0.4329**	**5**	**0.0866**			
Cor Total	**1130.28**	**59**				
Std. Dev	**1.39**					
Mean	**15.56**					
C.V. %	**8.95**					
R^2^	**0.9605**					
Adjusted R^2^	**0.8987**					
Predicted R^2^	**−2.3126**					
Adeq Precision	**19.1081**					

### Regression equation for 1‑OH‑PHZ production

The equation in terms of coded factors can be used to make predictions about the response for given levels of each factor. Here, the levels should be specified in the original units for each factor. This equation should not be used to determine the relative impact of each factor because the coefficients are scaled to accommodate the units of each factor, and the intercept is not at the center of the design space.

### Regression Equation for 1‑OH‑PHZ (2FI Model)

$$\begin{aligned}1-Hydroxyphe&nazine\\&=15.94874857492-0.35473010609A\\&+1.62773703397B+1.89335445231C\\&+1.86989351747D+1.55332669112E\\&+1.90256840147F+0.34092998269G\\&+0.47476672672H+0.95499665359 AB\\&-0.27262322913 AC-0.55747869655 AD\\&+0.58394046226 AE-0.69757258166 AF\\&+0.10275241170 AG+0.03193391102 AH\\&+1.04067585536 BC+1.00803132278 BD\\&+0.49310855771 BE-0.22786929636 BF\\&+0.39210845761 BG+0.57563690571BH\\&-0.88532498749 CD-1.13698513325 CE\\&-0.77350727918 CF+0.65801811793CG\\&-0.08201348125 CH+1.47440939933 DE\\&-0.47836274660 DF-0.66337641465 DG\\&-0.25311894867 DH+0.56318972705 EF\\&-0.66634269496 EG-0.02581847001 EH\\&+0.38459140732 FG+0.37151133757 FH\\&-0.37444850321 GH)\end{aligned}$$

### Response surface curve of 1‑OH‑PHZ Production

**Fig. 9:**
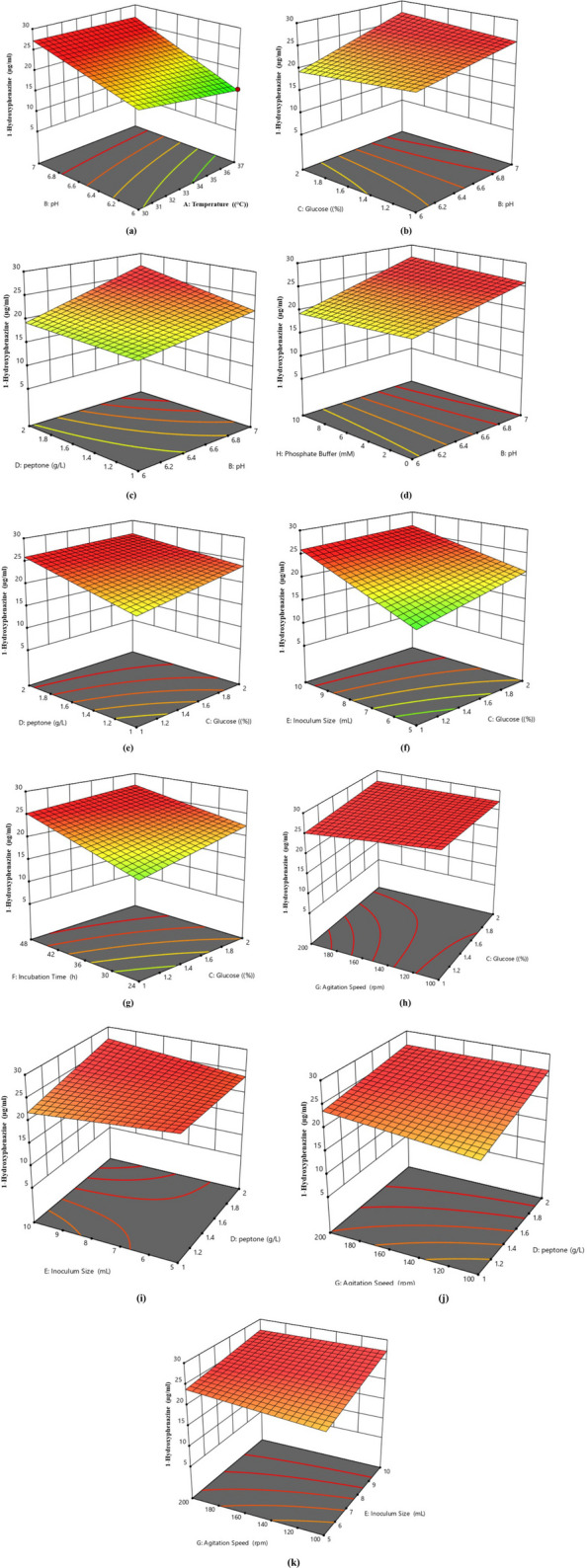
Response-surface plots illustrating the interactive effects of key physicochemical and nutritional factors on 1-hydroxyphenazine production by *P. aeruginosa* KAEH25. Panels (**a**–**k**) depict two-factor interactions generated using Response Surface Methodology (RSM): **a** Temperature × pH (AB), **b** pH × Glucose (BC), **c** pH × Peptone (BD), **d** pH × Phosphate Buffer (BH), **e** Glucose × Peptone (CD), **f** Glucose × Inoculum Size (CE), **g** Glucose × Incubation Time (CF), **h** Glucose × Agitation Speed (CG), **i** Peptone × Inoculum Size (DE), **j** Peptone × Agitation Speed (DG), and (**k**) Inoculum Size × Agitation Speed (EG)

The response-surface analyses (Fig. 9a–k) reveal the interactive effects of major nutritional and physicochemical variables on 1‑OH‑PHZ production by *P. aeruginosa* KAEH25. The response-surface analysis demonstrated that 1‑OH‑PHZ production was strongly influenced by interactions among nutritional and physicochemical parameters, with the most pronounced effects observed for pH, glucose concentration, peptone concentration, inoculum size, and incubation time. As shown in Fig. 9 (a–k), pH consistently exhibited a dominant positive influence across all interactions, with near-neutral conditions (pH ≈ 6.8–7.0) maximizing pigment synthesis regardless of the paired factor. Temperature synergized with pH to enhance production at higher levels (Fig. 9a), while glucose and peptone each showed significant cooperative effects with pH, producing elevated responses in Fig. 9b and c, respectively. Phosphate buffer contributed only a minor enhancement when combined with optimal pH (Fig. 9d). Interactions between carbon and nitrogen sources were strongly favorable, with glucose–peptone combinations yielding high pigment levels (Fig. 9e), and glucose also interacting positively with inoculum size (Fig. 9f) and incubation time (Fig. 9g) to promote late-phase secondary metabolite accumulation. By contrast, agitation speed showed minimal impact when paired with glucose (Fig. 9h), peptone (Fig. 9j), or inoculum size (Fig. 9k), indicating that aeration was not a limiting factor in the tested range. Peptone and inoculum size jointly exerted one of the strongest synergistic effects, producing steep response elevations at high levels of both variables (Fig. 9i). Collectively, the surfaces indicate that optimal 1-hydroxyphenazine biosynthesis is achieved under conditions in which pH, glucose, peptone, inoculum size, and incubation time converge at their upper effective ranges, while agitation and phosphate buffer exert comparatively minor influence.

### Antibacterial activity of 1‑OH‑PHZ

**Fig. 10:**
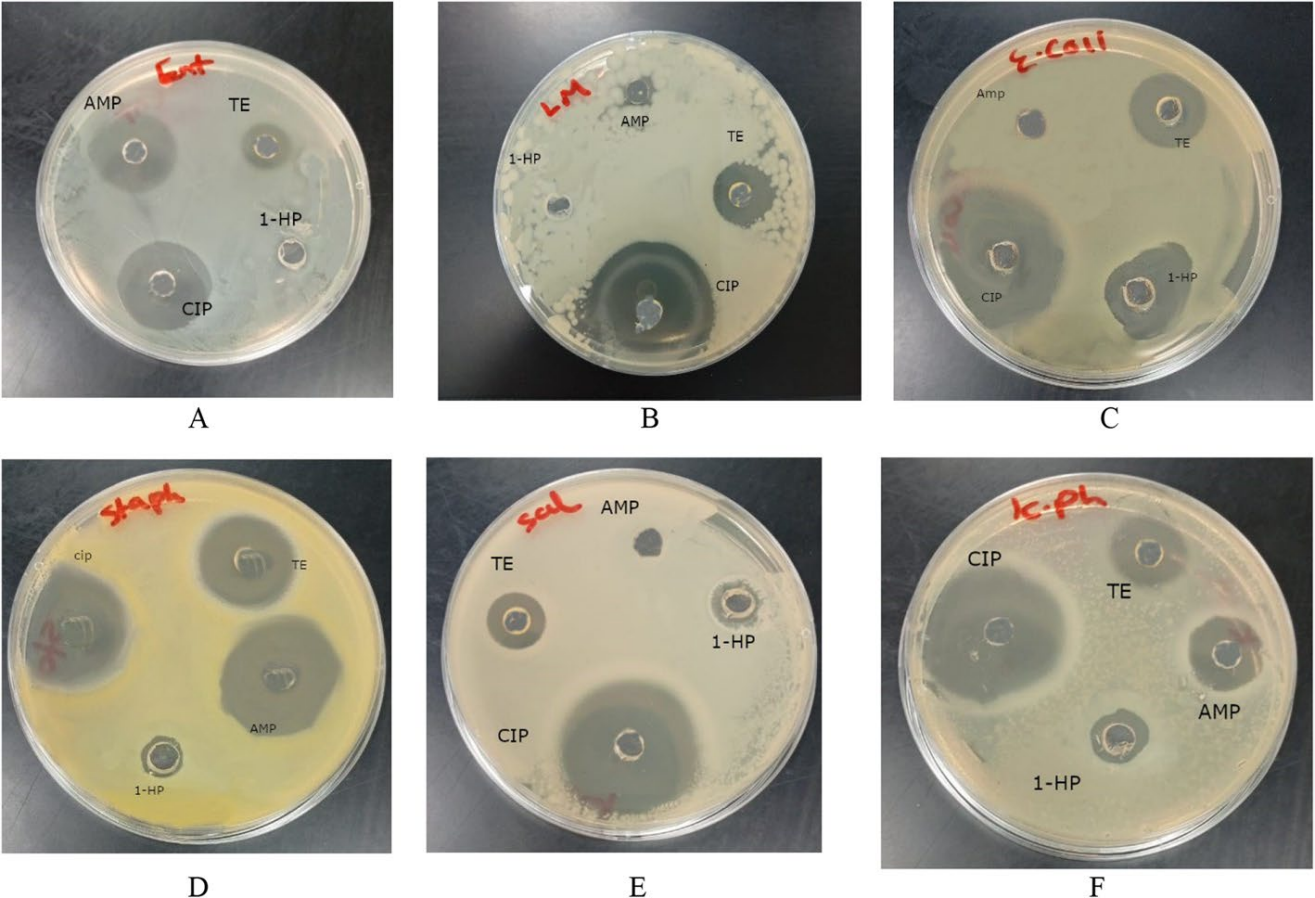
Antibacterial activity of 1‑OH‑PHZ and reference antibiotics against different bacterial strains using the Well diffusion method. **A** *E. faecalis*, **B** *L. monocytogenes*, **C** *E. coli* (**D**) *S. aureus*, (**E**) *S. enterica serovar Typhimurium*, **F** *K. pneumonia*

Preliminary agar well-diffusion assays demonstrated that 1-OH-PHZ possesses measurable antibacterial activity under the optimized production conditions (Table 9). 1‑OH‑PHZ exhibited clear antibacterial activity against four of the six tested pathogens. The compound produced inhibition zones of 20 ± 1 mm against *E. coli* ATCC 25922, 13 ± 0.5 mm against *S. enterica* serovar Typhimurium ATCC 14028, 13 ± 0.7 mm against *K. pneumoniae* DSM 25426, and 8 ± 0.3 mm against *S. aureus* ATCC 25923. No inhibition was observed for *E. faecalis* ATCC 29212 or *L. monocytogenes* ATCC 19114. The activity profile was reproducible across biological replicates and demonstrated selective potency, particularly toward Gram-negative species. Distinct and well-defined inhibition zones were observed across agar-Well diffusion assay plates (Fig. 10), confirming the antibacterial activity of 1‑OH‑PHZ. Clear circular zones of growth suppression were consistently produced against *E. coli* ATCC 25922, *S. enterica* serovar Typhimurium ATCC 14028, and *K. pneumoniae* DSM 25426. These visually pronounced inhibition halos correspond with the quantitative measurements reported and demonstrate selective activity toward Gram-negative pathogens. The substantial inhibition observed for *E. coli* in particular reinforces the compound’s potential as a promising lead scaffold for developing next-generation antimicrobial agents targeting high-risk bacterial pathogens. These results serve as *proof-of-concept evidence* confirming the bioactivity of purified 1-OH-PHZ and its selective activity toward Gram-negative bacteria.

**Table 9:** Zone of inhibition (mm, mean ± SD) of 1‑OH‑PHZ and reference antibiotics against tested bacteria

Bacterial strain	1‑OH‑PHZ	Ciprofloxacin	Tetracycline	Ampicillin
*S. enterica serovar Typhimurium ATCC 14028*	**13.0 ± 0.5**	**30.0 ± 0.4**	**12.0 ± 0.3**	**–**
*S. aureus* ATCC 25923	**8.0 ± 0.3**	**25.0 ± 0.5**	**20.0 ± 0.4**	**27.0 ± 0.6**
*E. coli* ATCC 25922	**20.0 ± 1.0**	**30.0 ± 0.3**	**18.0 ± 0.5**	**–**
*K. pneumoniae* DSM 25426	**13.0 ± 0.7**	**30.0 ± 0.4**	**15.0 ± 0.3**	**19.0 ± 0.5**
*E. faecalis* ATCC 29212	**–**	**25.0 ± 0.5**	**20.0 ± 0.4**	**12.0 ± 0.3**
*Listeria monocytogenes ATCC 19114*	**–**	**30.0 ± 0.3**	**15.0 ± 0.4**	**–**

(–) No inhibition zone observed

## Discussion

1-OH-PHZ, a microbial phenazine metabolite produced by *Pseudomonas* *aeruginosa*, exhibits broad-spectrum antibacterial activity and holds potential for plant disease management [4, 42, 43]. This compound is also recognized as a virulence factor, capable of generating reactive oxygen species. Recent advancements in genomics, structural biology, and bioprocess optimization offer robust avenues for enhancing its production. The integration of Nanopore sequencing, AlphaFold for enzyme structure prediction, and Plackett–Burman experimental design represents a synergistic, systems-level approach to rationally enhance 1-OH-PHZ biosynthesis in *P. aeruginosa*. This strategy offers a scalable, green alternative to synthetic bactericides in sustainable agriculture. Its dual role as both a defensive metabolite and a virulence-associated redox-active compound illustrates the complex ecological functions of phenazines in *Pseudomonas aeruginosa*. These properties underscore the importance of developing precise biosynthetic optimization strategies that enhance desirable activities while mitigating pathogenic attributes. The integrated genomic, structural, and bioprocessing approach applied in this study provides a coherent framework for rational enhancement of 1-OH-PHZ biosynthesis.

The taxonomic analysis of *P. aeruginosa* strain KAEH25 is strongly supported by its 16S rRNA sequence identity and phylogenetic placement. The 1,524-bp amplicon (GenBank: PX255551) showed ≥ 99.54% identity to multiple *P. aeruginosa* reference genomes, including CP174013, CP192623, MN547155, and OQ168635. In contrast, the nearest non-*aeruginosa* taxon, *P. fluorescens* SBW25, shared only 94.37% identity, a divergence well below accepted species-level thresholds and consistent with interspecies boundaries reported in related genera. Phylogenetic reconstruction further corroborated this classification, placing KAEH25 within a monophyletic *P. aeruginosa* clade with maximal (100%) bootstrap support [44, 45]. Although 16S rRNA analysis has recognized limitations in resolving strain-level differences [46, 47], the combination of high sequence identity, complete query coverage, and robust phylogenetic positioning provides unequivocal evidence that KAEH25 belongs to *P. aeruginosa* [44, 45]. This precise taxonomic resolution is essential, given the ecological and pathogenic relevance of the species.

The UV–Vis absorption spectrum of 1‑OH‑PHZ is characterized by a strong maximum at 261 nm, attributed to a π → π* transition within its conjugated aromatic system. This band reaches an absorbance of approximately 2.6, indicating a highly efficient electronic transition. A shoulder near 240 nm and weaker bands around 280 nm, with additional low-intensity features from 300–340 nm, are also detected [5]. These spectral elements are consistent with an extended π-conjugated framework that supports significant absorption in the UV–Vis region. The hydroxyl substituent at position 1 donates electron density into the chromophore, producing a red-shift and increasing the intensity of key transitions by promoting intramolecular charge transfer and narrowing the HOMO–LUMO gap [48, 49]. The shoulder at 240 nm is likely due to vibronic coupling or a higher-energy π → π* contribution [5]. A gradual decrease in absorbance from 280 to 400 nm indicates lower probabilities for electronic excitation at longer wavelengths. Weak n → π* or symmetry-forbidden transitions may contribute to the broad, low-intensity signals observed in the 300–340 nm region [5, 50]. These features align with established UV–Vis behavior of conjugated aromatic compounds, in which increased delocalization produces bathochromic shifts in λmax [51, 52]. The orange pigmentation observed on agar plates corresponds with the UV–Vis profile [5]. Absorption in the blue–violet region (approximately 400–470 nm) leads to the perception of orange light by reflection or transmission [3]. This color arises from electronic transitions within the phenazine chromophore, a core structural element of many microbial pigments [53]. Phenazines, including 1‑OH‑PHZ, function as redox-active secondary metabolites produced by several microorganisms, notably *Pseudomonas aeruginosa* [54, 55]. These spectral properties have practical significance for monitoring biosynthesis and for potential applications leveraging phenazine photophysics.

Nanopore sequencing provided high-resolution insight into the genomic organization of the phenazine biosynthetic locus in *P. aeruginosa* KAEH25, enabling complete reconstruction of the 10,358-bp region and precise annotation of *phzF–phzH*. The ability of long-read sequencing to span repetitive, GC-rich regions—limitations frequently encountered with short-read platforms—has been well documented [56]. In KAEH25, the high-quality dataset (1,222 reads; 2.3 Mbp; 62% GC) generated a single contiguous assembly, confirming the full operon structure along with flanking genes such as *fptX* and several hypothetical loci. These findings underscore the utility of Nanopore sequencing for resolving secondary-metabolite gene clusters, structural variants, and regulatory elements that influence phenazine biosynthesis [57, 58]. The comprehensive operon reconstruction provides a foundation for understanding metabolic capacity, regulatory architecture, and potential engineering targets in *P. aeruginosa*, supporting broader efforts to link genomic structure with phenotypic traits such as antimicrobial activity, virulence regulation, and biotechnological application of phenazines [59]. Integrating spectral characterization with genomic analysis illustrates the coherence between genotype and phenotype in KAEH25. The structurally intact phenazine operon, coupled with the measured optical signatures of 1‑OH‑PHZ, indicates that the strain synthesizes functional phenazine metabolites consistent with known biochemical pathways.

The biosynthesis of phenazines in *Pseudomonas aeruginosa*, including strain KAEH25, is governed by a conserved genetic locus that encodes the enzymes required for the stepwise conversion of chorismate-derived intermediates into phenazine-1-carboxylic acid (PCA) and its derivatives [60]. Genomic studies continue to demonstrate that this canonical gene organization is essential for efficient phenazine formation and for the ecological functions associated with these metabolites [17]. The core genes, *phzE*, *phzD*, *phzF*, *phzB*, and *phzG*, are positioned to enable the ordered transformation of chorismate into early phenazine intermediates [60]. Chorismate itself is a central metabolite within aromatic amino acid biosynthesis, emphasizing the integration of phenazine production within broader cellular metabolism [61]. In strain KAEH25, the gene order and orientation closely follow the established phenazine biosynthetic framework, supporting accurate and sequential enzymatic activity [17, 60]. Diversification of PCA into specialized phenazine derivatives is achieved through terminal tailoring enzymes. The presence of *phzH* and *phzS* in the KAEH25 locus indicates its capacity to synthesize phenazine-1-carboxamide and 1-OH-PHZ, respectively [7]. Hydroxylated phenazines, including 2-hydroxyphenazine, often show enhanced biological activity. Compounds such as 1-OH-PHZ display broad-spectrum antibacterial properties, while the flavin-dependent monooxygenase PhzO is able to convert PCA into 2-hydroxyphenazine, a compound with strong antifungal activity against *Gaeumannomyces graminis* var. *tritici* [11]. Phenazines, including PCA and pyocyanin, possess notable antimicrobial, antitumoral, and insecticidal activities [62, 63]. Several phenazines produced by *P. aeruginosa*, such as pyocyanin and PCA, contribute to virulence by generating reactive oxygen species and inducing oxidative stress in host cells [64]. Pyocyanin facilitates extracellular electron discharge under microaerobic and anaerobic conditions and inhibits a range of human pathogens [65]. PCA, also known as “Shenzimycin,” is widely recognized as an effective biocontrol agent and has been approved for agricultural use [66]. Efforts to modulate phenazine biosynthesis have been widely implemented in metabolic engineering. Deletions of *utr*, *phzM*, or *phzS* in *P. aeruginosa* M18 have been associated with increased PCA production [17, 66]. Since PhzM and PhzS convert PCA into pyocyanin, increasing their gene dosage can enhance pyocyanin yields [67]. PhzS also participates in the formation of 1-OH-PHZ [7]. Optimization strategies, including statistical experimental designs, have been used to define culture conditions, such as pH, temperature, and carbon sources, that maximize PCA production [68].

Structural predictions generated by InterProScan annotation and AlphaFold modeling add mechanistic depth to the genomic analysis and highlight that the seven phenazine-biosynthetic enzymes in *P. aeruginosa* KAEH25 adopt conserved structural folds characteristic of their catalytic functions, confirming a fully intact and operational phenazine biosynthetic pathway. The identification of canonical motifs—such as the TIM-barrel fold in PhzC, the isochorismatase fold in PhzD, the PLP-dependent aminotransferase architecture in PhzE, and the FMN- or FAD-binding Rossmann-like domains in PhzG and PhzS—underscores the evolutionary conservation of phenazine biosynthesis [7, 11, 69–71]. These structural insights align with recent experimental models demonstrating high concordance between AlphaFold predictions and crystallographic data for related Phz enzymes, reinforcing their reliability [11, 55, 72]. Importantly, resolving these enzymatic architectures provides a mechanistic framework for metabolic engineering, enabling targeted modification of rate-limiting steps and cofactor-binding sites to enhance phenazine yield [17, 66, 73]. Such structure-guided optimization is particularly valuable given the diverse bioactivities of phenazines and their growing relevance in biocontrol, antimicrobial development, and industrial biotechnology.

Statistical optimization using PBD and RSM provided insights into how environmental and nutritional factors shape phenazine biosynthesis. PBD has been effectively employed to identify key factors influencing the production of 1-OH-PHZ, a bioactive secondary metabolite with antifungal, anticancer, and insecticidal properties [5, 11, 74]. The factorial screening approach applied in this study, supported by Pareto-chart visualization and dual significance thresholds (Bonferroni limit = 7.70406; t-value limit = 3.18245), was used to distinguish highly significant from moderately significant process variables. Through this hierarchical analysis, peptone, pH, inoculum size, glucose concentration, incubation time, and temperature were identified as dominant contributors to 1-OH-PHZ production. These findings are consistent with the established view that phenazine biosynthesis in *Pseudomonas* spp. is regulated by nutritional and environmental cues, including carbon–nitrogen balance, culture pH, and growth-phase behavior [43, 75]. The ANOVA results demonstrated strong statistical robustness. A Model F-value of 256.34 (*p* = 0.04%) indicated that the variation in pigment yield was largely explained by the evaluated factors rather than experimental noise. Among the significant main effects, peptone (F = 672.25), pH (F = 432.98), and inoculum size (F = 426.14) exerted the greatest influence on 1-OH-PHZ production. The prominent effect of peptone concentration is mechanistically consistent with the dependence of phenazine biosynthesis on the shikimic acid pathway, which requires nitrogen-rich precursors such as chorismic acid. Peptone provides accessible nitrogen and amino acids that feed this pathway [42, 43]. Regulation by pH is attributed to its control over the protonation state of phenazine intermediates and the activity of regulatory elements within the *phz* gene cluster. Inoculum size influences the timing and strength of quorum-sensing activation, which is essential for phenazine expression because the *phz* operons are directly governed by N-acyl-homoserine lactone (AHL)–mediated signaling circuits [43]. The moderate significance of agitation speed, iron concentration, and phosphate buffer—factors that exceeded the t-value threshold but remained below the Bonferroni limit—requires careful interpretation rather than exclusion. Iron availability is particularly relevant because 1-OH-PHZ acts as a redox-active metabolite that reduces ferric to ferrous iron, influencing the iron-acquisition strategies of *P. aeruginosa* [42]. The redox cycling of phenazines, including 1-OH-PHZ, generates reactive oxygen species and alters the intracellular NADH/NAD⁺ ratio, establishing a feedback loop between iron metabolism and phenazine biosynthesis [43]. Agitation speed, by modulating dissolved oxygen levels, intersects with the function of 1-OH-PHZ as an electron shuttle, given that oxygen serves as the terminal electron acceptor during phenazine-mediated extracellular electron transfer. Phosphate buffer concentration shapes both the buffering environment and phosphate-responsive regulatory networks, such as the PhoB regulon, which can influence secondary-metabolite pathways. The model’s predictive capacity, reflected in a Predicted R^2^ of 0.9766 and an Adjusted R^2^ of 0.9946 (difference < 0.2), together with an adequate precision value of 48.883, confirms that meaningful signal was captured with minimal overfitting. The low residual error (0.3667) provides additional support for model adequacy. These diagnostic metrics collectively validate the suitability of the model for exploring the design space in subsequent optimization phases, including central composite or Box–Behnken designs for response-surface modelling. Recent studies in phenazine bioproduction highlight substrate engineering as a complementary strategy. Pantelić et al. (2023) demonstrated that food-waste substrates—particularly stale bread—can support *P. aeruginosa* growth and yield purified 1-OH-PHZ at approximately 4.4 mg L⁻^1^ after 24 h in 5-L bioreactors, linking medium optimization with circular-bioeconomy goals [5]. Similarly, Nguyen et al. (2022) showed that fishery-processing by-products (e.g., squid pens) influence phenazine yield and composition, underscoring the importance of carbon and nitrogen composition in complex substrates [76]. These findings support the present factorial results, confirming that nitrogen-source identity (peptone) and carbon availability (glucose) are among the most influential variables. From a translational standpoint, the statistical framework established here is well aligned with renewed interest in 1-OH-PHZ as a therapeutic scaffold. Abuelhaded et al. (2025) positioned 1-OH-PHZ within antimicrobial-resistance research and synthetic-biology applications, noting that metabolic engineering and nanocarrier-based systems have enabled scalable production with reduced host toxicity [42]. The ability to identify and rank production-determining variables through factorial screening provides a statistical foundation for such scale-up efforts. Ali et al. (2025) further confirmed that optimized culture conditions (35 °C, pH 7, glucose and casein as carbon and nitrogen sources) enhance pigment production in environmental *P. aeruginosa* isolates, findings that are consistent with the hierarchy revealed in this study [75].

The iterative nature of experimental design, from screening with PBD to optimization with RSM, is a fundamental strategy in biomanufacturing to achieve high yields and robustness [77]. This methodical approach ensures that resources are efficiently allocated to variables with the greatest impact, facilitating the development of sustainable and cost-effective bioproduction systems [78].

The biosynthesis of 1‑OH‑PHZ by *P. aeruginosa* strain KAEH25 is influenced by multiple nutritional and environmental factors, as demonstrated through the RSM framework. The CCD matrix of 60 experimental runs indicates that 1‑OH‑PHZ biosynthesis is driven by complex, nonlinear interactions among cultivation parameters rather than by any single dominant factor. The production range of 5.44–24.85 µg/mL across the design space highlights the high sensitivity of phenazine biosynthesis to the combined effects of nutritional, physicochemical, and temporal variables. This pattern is consistent with evidence that phenazine yields in *Pseudomonas* spp. are closely regulated by environmental cues influencing secondary-metabolite pathways [43]. Higher 1‑OH‑PHZ concentrations were consistently associated with near-neutral pH values (approximately 6.5–7.0), moderate glucose levels (1–2%), elevated peptone concentrations, and extended incubation periods. This pattern aligns with established principles of phenazine regulation in pseudomonads, where biosynthetic gene clusters—particularly the *phzABCDEFG* core operon—are activated in a quorum-sensing–dependent manner and are strongly influenced by nutrient availability and growth-phase dynamics [43, 66]. The positive effect of peptone on 1‑OH‑PHZ output likely reflects the nitrogen-dependent regulation of the shikimate pathway, which supplies chorismic acid as the key precursor for phenazine ring assembly. Engineering strategies that enhance shikimate-pathway flux in *P. chlororaphis* Lzh-T5 have yielded substantial increases in phenazine-1-carboxylic acid (PCA) production, from 230 mg/L to 10,653 mg/L, demonstrating the central importance of precursor supply as a rate-limiting step [66]. Although PCA represents a distinct phenazine derivative, it functions as the immediate precursor of 1‑OH‑PHZ through the monooxygenase PhzS, indicating that factors enhancing PCA flux are expected to exert upstream control over 1‑OH‑PHZ biosynthetic capacity [7]. The detrimental effect of low pH on 1‑OH‑PHZ production, observed in several CCD runs (e.g., Run 37, pH 5.66, yielding 10.78 µg/mL), is consistent with the pH sensitivity of phenazine regulatory networks. Response-surface optimization of pyocyanin, another Pseudomonas-derived phenazine, has similarly identified pH as a critical determinant, with optimal values clustering near neutrality [53]. The moderating role of agitation speed—which displayed a non-monotonic relationship with 1‑OH‑PHZ output across the design space (e.g., Run 15 at 65.9 rpm producing 15.81 µg/mL vs. Run 55 at 234.1 rpm producing 17.91 µg/mL)—likely reflects the oxygen dependence of the hydroxylation step catalyzed by PhzS, balanced against potential shear-related impacts on cellular physiology [7]. The maximum 1‑OH‑PHZ concentration obtained in this CCD (24.85 µg/mL) should be considered in the context of recent benchmarks in phenazine bioproduction. Metabolic engineering of *P. chlororaphis* H18 through heterologous expression of *PhzS, NaphzNO1*, and *LaphzM* has been used to diversify 1-hydroxyphenazine derivatives, although absolute titers have remained limited by monooxygenase efficiency and cofactor availability [53]. Fermentation of *P. aeruginosa* BK25H on food-waste substrates, such as stale bread, has yielded approximately 4.4 mg/L of purified 1‑OH‑PHZ after 24 h of batch cultivation [5]. Cassava-starch processing by-products have also been investigated as cost-effective carbon sources for 1‑OH‑PHZ production in *P. aeruginosa* [8]. The lower concentrations observed in the present CCD may reflect inherent strain differences, extraction variability, or the design of the RSM matrix, which intentionally includes suboptimal regions of the factor space to enable accurate polynomial model fitting. For the related derivative phenazine-1-carboxamide (PCN), optimization of *P. chlororaphis* H5△fleQ△relA by sequential Plackett–Burman and response-surface designs have achieved titers of 5.51 ± 0.17 g/L [79], demonstrating gram-per-liter production levels that exceed current 1‑OH‑PHZ capabilities. This disparity highlights a central constraint: in contrast to PCA or PCN, 1‑OH‑PHZ biosynthesis depends on a dedicated hydroxylation step whose catalytic efficiency remains a major bottleneck. Work on the analogous compound 2-hydroxyphenazine (2-OH-PHZ) has shown that the low activity of the flavin-dependent monooxygenase PhzO, along with limited cofactor regeneration and suboptimal process parameters, represents a key barrier to productivity. An integrated cofactor–pathway–process engineering strategy was developed to overcome these limitations for 2-OH-PHZ, resulting in markedly improved titers in *P. chlororaphis* [12]. The two-factor interaction (2FI) model developed here captures the dominant nutritional and physicochemical determinants of 1-OH-PHZ biosynthesis by *P. aeruginosa* KAEH25, yet its diagnostic statistics reveal important methodological tensions that merit careful interpretation before the biological findings can be contextualized. The model’s high R^2^ value (0.9605) indicates strong explanatory capacity, and the adequate precision of 19.11, well above the threshold of 4, demonstrates a sufficient signal-to-noise ratio for navigating the design space. However, the pronounced discrepancy between the adjusted R^2^ (0.8987) and the predicted R^2^ (− 2.3126) provides a critical diagnostic warning. A negative predicted R^2^ indicates that the overall mean predicts new observations more effectively than the fitted model, reflecting an overfitting scenario in which noise is captured instead of true process structure [80]. This pattern—characterized by excellent training performance but poor generalization—is well known in predictive-modelling research and typically arises when the number of model terms approaches the number of experimental runs [80, 81]. With 36 parameters (8 linear terms and 28 interaction terms) fitted to 60 observations, the present design approaches the parametric density where overfitting becomes likely. Robust validation approaches, including leave-one-out or *k*-fold cross-validation, are therefore required to distinguish genuine predictive performance from artefact [82, 83]. The significant lack of fit (F = 28.36; *p* = 0.0008) further supports the conclusion that the 2FI model, while capable of capturing first-order interactions, omits higher-order curvature that likely governs the response near optimal conditions. Despite these statistical limitations, the ANOVA results provide biologically coherent insights into 1-OH-PHZ production. The five dominant linear factors—pH (F = 50.70), glucose (F = 51.84), peptone (F = 50.56), inoculum size (F = 32.02), and incubation time (F = 45.68)—reflect the established principle that phenazine biosynthesis functions as a secondary metabolic process regulated by nutrient availability, population density, and culture-phase transitions [84, 85]. The strong performance of near-neutral pH (≈ 6.8–7.0) is consistent with the pH sensitivity of the *phz* operon and the flavin-dependent monooxygenases PhzS and PhzM, which catalyze the terminal hydroxylation and methylation reactions in phenazine diversification [13, 86]. These enzymes require precise protonation states for optimal catalysis, and deviations from neutrality likely impair both enzyme activity and precursor availability, including chorismate and PCA. The significant glucose–peptone interaction (F = 10.70; *p* = 0.0034) and the individual importance of each factor further highlight the centrality of carbon–nitrogen balance in phenazine regulation. PCA, the universal precursor of all decorated phenazines including 1-OH-PHZ, is synthesized via the shikimate pathway, which competes with central carbon metabolism for phosphoenolpyruvate and erythrose-4-phosphate [84, 85]. Excess glucose may trigger catabolite repression and suppress secondary metabolism, whereas peptone provides nitrogen and amino acids required for chorismate formation. The cooperative effect observed here suggests that balanced C:N ratios direct metabolic flux toward the shikimate pathway while avoiding repression, aligning with bioprocess principles in which titer, rate, and yield must be balanced against metabolic burden. The pronounced inoculum-size and peptone interaction (F = 26.29; *p* < 0.0001) represents the strongest pairwise effect in the model and is notable from a regulatory perspective. Phenazine biosynthesis in *Pseudomonas* spp. is governed by quorum-sensing (QS) circuits in which population density drives autoinducer accumulation and subsequent activation of the *phz* operons [42]. Larger inoculum volumes accelerate the attainment of QS-activation thresholds, while high peptone concentrations sustain the elevated cell densities required to maintain autoinducer levels above this threshold throughout the production phase. This synergistic interaction has direct implications for scale-up, where inoculum density and nitrogen-feeding strategies must be co-optimized to synchronize QS activation with nutrient supply. Such coordination has been demonstrated in recent engineering efforts in *P. chlororaphis* H18, where integrated pathway and regulatory manipulation enabled 1-OH-PHZ titers of 3.6 g/L [13]. The significance of incubation time and its interactions with glucose (F = 6.92) and inoculum size reflects the temporal dynamics of secondary metabolism. Accumulation of 1-OH-PHZ occurs primarily during late-phase growth, requiring early biomass formation before metabolic flux is redirected from primary to secondary pathways [84]. The glucose and incubation-time interaction likely captures the metabolic shift from growth-associated glucose consumption to stationary-phase phenazine synthesis, a transition that has been strategically leveraged in two-stage fermentation systems developed for engineered *Pseudomonas* strains [7]. The non-significance of temperature ($F=1.70$; $p=0.2057$) and agitation speed ($F=1.42$; $p=0.2450$) within the tested ranges suggests that these physical parameters were already near-optimal or that their effects are overshadowed by nutritional regulation under the experimental conditions. However, this interpretation must be tempered by the limited range tested; RSM results are inherently local approximations, and broader factor ranges might reveal significant effects, particularly for agitation, which governs dissolved oxygen — a known modulator of phenazine redox cycling and reactive oxygen species generation [42, 85].

The strong alignment between experimental results and model predictions highlights RSM as an effective tool for delineating the complex interactions regulating 1-OH-PHZ biosynthesis [87]. These findings reinforce that balanced carbon and nitrogen sources, controlled pH, and optimized inoculum density are central to maximizing phenazine output [68]. Similar optimization principles have guided improvements in other microbial bioprocesses, including polygalacturonase [88], keratin hydrolysates [89], and chitooligosaccharides [90]. The growing use of low-cost carbon sources, such as agricultural residues, further illustrates the scalability of these approaches [91]. Phenazine production in *P. aeruginosa* is tightly regulated by nutrient availability and quorum-sensing networks [92]. The present findings support this relationship and provide a foundation for rational enhancement of phenazine biosynthesis. Integration of RSM optimization with population genomics and targeted metabolic engineering, as demonstrated in *P. chlororaphis* [26], represents a promising direction for further improving 1-OH-PHZ yield. Systems-level approaches such as flux balance analysis could also assist in identifying metabolic bottlenecks and guiding engineering strategies aimed at increasing pathway flux toward 1-OH-PHZ production.

The biological significance of 1-OH-PHZ is further strengthened by its demonstrated antibacterial activity. 1-OH-PHZ has demonstrated significant and selective antibacterial activity, particularly against Gram-negative bacteria. In agar well-diffusion assays, notable inhibition zones were produced against *E. coli* ATCC 25922 (20 ± 1 mm), *S. enterica* serovar Typhimurium ATCC 14028 (13 ± 0.5 mm), and *K. pneumoniae* DSM 25426 (13 ± 0.7 mm). This activity is considered important because multidrug-resistant (MDR) Gram-negative infections continue to increase and are projected to cause up to 10 million deaths annually by 2050 [42]. Moderate inhibition was observed against the Gram-positive strain *S. aureus* ATCC 25923 (8 ± 0.3 mm), while no activity was detected against *E. faecalis* ATCC 29212 or *L. monocytogenes* ATCC 19114. The selective inhibition of Gram-negative pathogens—including *E. coli*, *S. enterica*, and *K. pneumoniae*—is consistent with the ability of hydroxylated phenazines to penetrate the outer membrane and exert redox-mediated respiratory disruption.Increasing interest in phenazines has been driven by the urgent need for new antimicrobial agents in the face of rising antibiotic resistance [4]. Mechanistic studies have shown that hydroxylated phenazines, including 1-OHPZ, disrupt bacterial respiration through electron shuttling and promote reactive oxygen species (ROS)-mediated DNA damage [42]. These redox-cycling mechanisms distinguish phenazines from many conventional antibiotics and position them as promising scaffolds for drug development, especially as resistance to frontline agents continues to increase [93]. Their relevance has been highlighted by the continued emergence of resistance mechanisms such as target-site mutations, efflux pump overexpression, and enzymatic inactivation [42]. Additional research has reported antibacterial and antibiofilm activity from quinazoline derivatives [94] and DNA-gyrase inhibition by other novel scaffolds [95]. The formation of clear, well-defined inhibition zones by 1-OHPZ supports its effective diffusion and interaction with bacterial cells. The strong inhibition observed against *E. coli* ATCC 25922 underscores the potential of 1-OHPZ as a lead scaffold for next-generation therapeutics targeting high-risk Gram-negative pathogens [42]. This trend parallels efforts to identify compounds with mechanisms distinct from those of existing antibiotics, as resistance continues to expand across clinical settings [96]. Compounds such as the norfloxacin derivative NF22 have also shown activity against both standard and MDR *E. coli* strains [97]. The phenazine scaffold offers chemically accessible positions, particularly at C-1 and N-5, enabling medicinal modifications that may enhance pharmacokinetics while retaining Gram-negative targeting properties [42]. Additional antibacterial scaffolds have been explored, including dihydrophenazines active against *Acinetobacter baumannii* [98] and several hydrazone- and triazole-based derivatives [99, 100]. Nanomaterials such as chitosan–polyacrylic acid nanoparticles and metal-oxide nanocomposites have also been evaluated for antimicrobial and antibiofilm activities [101]. These diverse strategies reflect the global, multi-platform effort to develop new agents capable of countering antimicrobial resistance [102]. The performance of 1-OH-PHZ in the present work aligns with these broader research trends and underscores its potential as a tunable antimicrobial lead.

Collectively, the integration of genomics, structural biology, and statistical bioprocess engineering establishes a powerful systems-level strategy for improving phenazine biosynthesis. This multi-layered approach is well positioned to support metabolic engineering initiatives aimed at enhancing yield, modulating redox activity, and reducing virulence-associated effects. Given the ecological importance of *P. aeruginosa*, the agricultural value of phenazines, and the global need for novel antimicrobial scaffolds, such strategies hold considerable promise for advancing both biocontrol and therapeutic applications.

## Conclusion

This study demonstrates that integrating long-read genomics, high-accuracy structural modeling, and statistical bioprocess optimization provides an effective and scalable strategy for enhancing 1-OH-PHZ production in *Pseudomonas aeruginosa* KAEH25. The combined approach substantially increased pigment yield and confirmed the structural integrity and functional coherence of the phenazine biosynthetic pathway. The optimized process parameters produced markedly higher titers, while preliminary antimicrobial assays verified the compound’s selective activity against clinically relevant Gram-negative bacteria. These findings highlight the value of a systems-level framework for improving microbial secondary metabolites and underscore the potential of 1-OH-PHZ as a promising lead for future antimicrobial development. Moving forward, expanding this strategy through metabolic engineering, scale-up studies, and integration of MIC-based potency assessments will further advance the biotechnological and therapeutic potential of this compound.

## Supplementary Information

Supplementary Material 1. Design expert – Plackett–Burman design model.

Supplementary Material 2. Design expert - Central-composite design (CCD).

## Data Availability

The data supporting the findings of this study, including the 16S rRNA sequence (GenBank accession number PX255551) and raw FASTQ files of phenazine biosynthetic genes for Pseudomonas aeruginosa strain KAEH25, are available from the corresponding author, Ahmed H. I. Faraag ([professor_ahmed85@science.helwan.edu.eg](mailto:professor_ahmed85@science.helwan.edu.eg)), upon reasonable request.
